# Exosomes From Human Cardiac Progenitor Cells for Therapeutic Applications: Development of a GMP-Grade Manufacturing Method

**DOI:** 10.3389/fphys.2018.01169

**Published:** 2018-08-24

**Authors:** Gabriella Andriolo, Elena Provasi, Viviana Lo Cicero, Andrea Brambilla, Sabrina Soncin, Tiziano Torre, Giuseppina Milano, Vanessa Biemmi, Giuseppe Vassalli, Lucia Turchetto, Lucio Barile, Marina Radrizzani

**Affiliations:** ^1^Lugano Cell Factory, Fondazione Cardiocentro Ticino, Lugano, Switzerland; ^2^Swiss Institute for Regenerative Medicine, Torricella-Taverne, Switzerland; ^3^Division of Cardiac Surgery, Fondazione Cardiocentro Ticino, Lugano, Switzerland; ^4^Laboratory of Molecular and Cellular Cardiology, Fondazione Cardiocentro Ticino, Lugano, Switzerland; ^5^Department of Heart and Vessels, Laboratory of Cardiovascular Research, Lausanne University Hospital, Lausanne, Switzerland; ^6^Division of Cardiology, Fondazione Cardiocentro Ticino, Lugano, Switzerland

**Keywords:** exosomes, cardiac progenitor cells, good manufacturing practices, large-scale production, quality control

## Abstract

Exosomes, nanosized membrane vesicles secreted by cardiac progenitor cells (Exo-CPC), inhibit cardiomyocyte apoptosis under stress conditions, promote angiogenesis *in vitro*, and prevent the early decline in cardiac function after myocardial infarction *in vivo* in preclinical rat models. The recognition of exosome-mediated effects has moved attempts at developing cell-free approaches for cardiac repair. Such approaches offer major advantages including the fact that exosomes can be stored as ready-to-use agents and delivered to patients with acute coronary syndromes. The aim of the present work was the development of a good manufacturing practice (GMP)-grade method for the large-scale preparation of Exo-CPC as a medicinal product, for a future clinical translation. A GMP-compliant manufacturing method was set up, based on large-scale cell culture in xeno-free conditions, collection of up to 8 l of exosome-containing conditioned medium and isolation of Exo-CPC through tangential flow filtration. Quality control tests were developed and carried out to evaluate safety, identity, and potency of both cardiac progenitor cells (CPC) as cell source and Exo-CPC as final product (GMP-Exo-CPC). CPC, cultured in xeno-free conditions, showed a lower doubling-time than observed in research-grade condition, while producing exosomes with similar features. Cells showed the typical phenotype of mesenchymal progenitor cells (CD73/CD90/CD105 positive, CD14/CD20/CD34/CD45/HLA-DR negative), and expressed mesodermal (TBX5/TBX18) and cardiac-specific (GATA4/MESP1) transcription factors. Purified GMP-Exo-CPC showed the typical nanoparticle tracking analysis profile and expressed main exosome markers (CD9/CD63/CD81/TSG101). The GMP manufacturing method guaranteed high exosome yield (>10^13^ particles) and consistent removal (≥97%) of contaminating proteins. The resulting GMP-Exo-CPC were tested for safety, purity, identity, and potency *in vitro*, showing functional anti-apoptotic and pro-angiogenic activity. The therapeutic efficacy was validated *in vivo* in rats, where GMP-Exo-CPC ameliorated heart function after myocardial infarction. Our standardized production method and testing strategy for large-scale manufacturing of GMP-Exo-CPC open new perspectives for reliable human therapeutic applications for acute myocardial infarction syndrome and can be easily applied to other cell sources for different therapeutic areas.

## Introduction

Exosomes are EV of endosomal origin, with a diameter of 30–150 nm, involved in intercellular communication processes. They are secreted by virtually all cell types and express characteristic exosomal markers, such as CD9, CD63, CD81, Alix, and TSG101, as well as markers typical of the cells from which they originate. Cell origin is also a determinant for the microRNA content (Thery et al., 2006; Cervio et al., 2015; Kowal et al., 2016; Barile et al., 2017b; Sluijter et al., 2018; Van Niel et al., 2018).

Exo are currently the focus of various studies aimed at clarifying their role, mechanisms of action, and potential diagnostic and therapeutic applications (Lener et al., 2015; Barile and Vassalli, 2017).

Recent evidence suggests that human CPC release EV that phenotypically recapitulate Exo (Barile et al., 2012). Such vesicles inhibit cardiomyocyte apoptosis and promote angiogenesis *in vitro*, hence playing a fundamental role in the paracrine effect of CPC. *In vivo*, in a preclinical model of AMI in rats, CPC-derived Exo (Exo-CPC) reduce tissue damage, inhibit cardiomyocyte apoptosis, and promote angiogenesis, improving post-infarction cardiac function (Barile et al., 2014). Key mediators of these effects have been identified in specific microRNAs such as miR-132 and miR-210 (Barile et al., 2014), as well as specific proteins such as the active form of pregnancy-associated plasma protein-A (Barile et al., 2018).

Based on the above-described functional properties, Exo-CPC represent a promising candidate for future clinical applications, relying on an alternative and innovative approach in respect to cellular therapies currently being tested in the cardiovascular field. Compared to a cellular product, isolated Exo could have advantages in terms of safety and ease of handling, thanks to their low toxicity, high stability, biocompatibility, and biological barrier permeability (Xitong and Xiaorong, 2016). Moreover, Exo demonstrate strong potential for immunomodulation by inhibition of lymphocyte proliferation (Monguio-Tortajada et al., 2017) and reduction of CD68^+^ infiltrating cells once injected into heart (De Couto et al., 2017). Thus, Exo can be conceived for both autologous and allogeneic applications. This would be particularly useful in a context of AMI where the allogeneic use would allow the availability of an off-the-shelf Exo-based product, ready to be infused during percutaneous intervention to open occluded vessel as supporting cardioprotective therapy.

Exo from different cell types are being tested in clinical trials in several therapeutic areas, including cancer, diabetes, wound healing (Lener et al., 2015; Barile and Vassalli, 2017; Sluijter et al., 2018). Phase I–II immunotherapy studies for colon rectal cancer, melanoma and non-small cell lung cancer have already been completed with dendritic cell-derived Exo, demonstrating safety and feasibility of the approach; partial clinical responses were also detected in some patients (Pitt et al., 2016).

Several methods for EV and Exo isolation have been described, based on different specific characteristics, such as density, size, shape, and surface markers. Differential centrifugation/ultracentrifugation, polymer-based precipitation, filtration, size exclusion chromatography, density-gradient centrifugation, and magnetic bead capture are the most popular technologies according to a recent survey (Gardiner et al., 2016). However, there is not yet a state-of–the-art method for the production of large-scale clinical-grade EV, a context in which scalability, reproducibility, safety, potency, and purity of the resulting product represent crucial issues.

In this context, the development and validation of GMP-grade methods to produce and functionally validate Exo is essential for a future clinical translation.

The aim of the present work was the development of production and QC methods for the large-scale preparation of Exo-CPC as a medicinal product, in accordance with current GMP (GMP-Exo-CPC).

The starting point for such development were the research grade methods used so far for CPC isolation and culture as well as for small-scale production of Exo-CPC for pilot preclinical experiments (Barile et al., 2014, 2018). In such basic research context, open culture vessels (Petri dishes) and poorly standardized, animal-derived components (FBS, gelatin, and trypsin) have been used for CPC culture and expansion. Exo-CPC were isolated by polymer-based precipitation or ultracentrifugation, both hardly scalable and potentially favoring the concomitant concentration of impurities. Moreover, both CPC and Exo-CPC were poorly characterized, in terms of safety (e.g., sterility) and purity aspects (presence of process-derived contaminants), and the applied characterization and functional assays were unsuitable for formal validation.

The approach followed throughout the present work was focused on overcoming these limitations: the use of open culture vessels was avoided as much as possible, xeno-free reagents were selected as alternatives to the animal-derived ones, and reliable and scalable isolation methods were tested. Moreover, the analytical methods have been revisited and optimized in order to achieve the reproducibility and robustness required for their application in a GMP context.

Finally, CPC and GMP-Exo-CPC were extensively checked for identity, purity, safety, and functional activity.

## Materials and Methods

Cardiac progenitor cells and Exo-CPC production methods are described in detail in this section. Analytical methods are outlined here and reported in detail in **Supplementary Materials**.

### CPC Isolation and Culture

Atrial appendage explants were obtained from patients subjected to valve repair surgery who were not carriers of concomitant coronary artery disease (*n* = 9, seven males and two females; age 69 ± 7 years; LVEF 61 ± 6%).

Cardiac tissue specimens were stored in a sterile vessel containing cardioplegic solution [Plasma-Lyte A^®^ solution (Baxter Healthcare, United States) supplemented with Mannitol (final concentration 0.3%), magnesium sulfate (0.2%), sodium bicarbonate (0.1%), lidocaine (0.01%), and potassium chloride (0.2%), all from Sigma-Aldrich/Merck, United States] and transferred to labs. The tissue was processed under sterile conditions (Class A laminar hood): after transfer to a sterile support (Petri dish, Corning, United States) two washings were performed with Dulbecco’s phosphate buffered saline without calcium and magnesium (DPBS, Gibco/Thermo Fisher Scientific, United States), then the cardiac muscle tissue was isolated from the connective tissue and minced in small fragments (around 1 mm diameter).

#### Research-Grade Process

Tissue fragments were placed to adhere on gelatin (Sigma-Aldrich/Merck, United States)-coated 10 μm Petri dishes (Corning), in the presence of IMDM culture medium (Lonza, Switzerland) supplemented with 20% FBS (Gibco/Thermo Fisher Scientific), then incubated at 37°C in 5% CO_2_. After few days, CPC outgrowth was observed. At confluence, CPC were harvested through trypsin (Sigma-Aldrich/Merck) treatment, then seeded at 8–10 × 10^4^ cells/cm^2^ in appropriate flasks and expanded in the same culture conditions (see **Table 1**). The flasks were previously coated in presence of gelatin solution 0.2% in DPBS, incubated for 30 min at RT.

**Table 1:** Different methods for CPC isolation and culture.

		GMP grade I (CPC-I)	GMP grade II (CPC-II)	RESEARCH grade (CPC-R)
CPC isolation	Adhesion substrate	CELLstart^TM^ CTS^TM^	Synthemax^®^ II-SC	Gelatin
	Culture medium	StemMACS-MSC expansion Media kit XF		IMDM 20% FBS
	Dissociation enzyme	TrypLE^TM^ Select		Trypsin
CPC expansion	Adhesion substrate	CellBIND^®^ surface		Gelatin
	Culture medium	StemMACS-MSC expansion Media kit XF		IMDM 20% FBS
	Dissociation enzyme	TrypLE^TM^ Select		Trypsin

CPC expanded in these research grade conditions are thereafter indicated as CPC-R.

#### Setup of GMP-Grade Process

Tissue fragments were placed in 115 cm^2^ tissue-culture flasks with reclosable lids (TPP, Switzerland) in the presence of StemMACS-MSC expansion Media kit XF (Miltenyi Biotec GmbH, Germany) after coating with CELLstart^TM^ CTS^TM^ (Gibco/Thermo Fisher Scientific) or Synthemax^®^ II-SC (Corning) as adhesion substrates for “GMP grade I” and “GMP grade II” conditions, respectively (see **Table 1**). For the coating with CELLstart^TM^ CTS^TM^, flasks were incubated with the reagent diluted 1:50 in DPBS for 2 h at 37°C. Regarding the coating with Synthemax^®^ II-SC, flasks were incubated with the reagent diluted 1:40 in sterile water for 2 h at RT.

At confluence, CPC were harvested using TrypLE^TM^ Select Enzyme (Gibco/Thermo Fisher Scientific), then seeded at 8–10 × 10^4^ cells/cm^2^ and expanded in T flasks (75–150 cm^2^), HYPERFlask^®^ (1720 cm^2^) or HYPERStack^®^-12 (6000 cm^2^) culture vessels (Corning). Thanks to their negatively charged, highly hydrophilic CellBIND^®^ surface (Corning), designed to facilitate cell attachment and spreading, HYPERFlasks and HYPERStacks did not require any coating.

For CPC MCB and PPCB generation (see below), the above indicated StemMACS-MSC expansion Media kit XF was substituted by its GMP counterpart, MSC-Brew GMP Medium (test lot kindly provided by Miltenyi Biotec GmbH).

CPC expanded in GMP grade conditions I and II are thereafter indicated as CPC-I and CPC-II, respectively.

### Conditioned Medium Preparation

Cardiac progenitor cells expanded up to culture passage 4 (P4) were washed twice with DPBS and incubated at 37°C with 5% CO_2_ in Dulbecco’s Modified Eagle Medium (DMEM) 4.5 g/l glucose without phenol red (Gibco/Thermo Fisher Scientific, catalog number 21063-029), thereafter indicated as production medium. After 1–2 weeks, the CM was harvested.

### Exosome Isolation

Three different methods were used throughout this study for Exo-CPC isolation.

#### Ultracentrifugation

Serial centrifugation/ultracentrifugation steps were applied, as previously described (Barile et al., 2018). Briefly, 15 ml of CM were centrifuged at 3000 *g*, 4°C, 15 min; the supernatant was collected, filtered (0.2 μm filter, TPP), transferred in Amicon Ultra-15 (100 kDa cut-off, Merck Millipore, Germany) and centrifuged at 3000 *g*, 4°C, for 30 min; the concentrate was centrifuged at 10,000 *g*, 4°C, for 20 min; the supernatant was collected, diluted to 3 ml with DPBS and ultracentrifuged at 100,000 *g*, 4°C, for 120 min; the resulting pellet was suspended in 100 μl of DPBS.

This method was exclusively used for small-scale Exo isolation, to compare Exo derived from CPC cultured in the different conditions reported in **Table 1**.

#### Direct Filtration

The CM was clarified by centrifugation at 3000 *g*, 15 min at RT, followed by filtration (0.22 μm) in bottle filter units (TPP). Concentration was performed by direct filtration through Amicon Ultra-15 (100 kDa cut-off) (15 ml CM/device) or Centricon Plus-70 (100 kDa cut-off, Merck Millipore) (65 ml CM/device), centrifuged at 3000 *g*, RT, 30 min. Each resulting concentrated sample was diluted about 1:10 with Plasma-Lyte A^®^ solution and concentrated again using the same device.

This method was applied during process scale up.

#### Tangential Flow Filtration (TFF)

The CM was clarified by 0.22 μm filtration through bottle filter units or online filters (ULTA Capsule HC, KMP-HC9202HH, GE Healthcare, United States). Concentration was performed by TFF, using the ÄKTA^TM^ flux 6 system (GE Healthcare) equipped with a 300 kDa cut-off hollow fiber cartridge (GE Healthcare); the concentration was followed by diafiltration in five volumes of Plasma-Lyte A^®^ solution. Process details are reported below.

This method was applied during process scale up and for the large-scale manufacturing of GMP-Exo-CPC.

### CPC Master Cell Bank and Post-production Cell Bank

Cardiac progenitor cells were isolated and cultured according to the “GMP grade I” method described above, using CELLstart^TM^ CTS^TM^ as adhesion substrate and MSC-Brew GMP Medium as culture medium.

At culture passage 2 (P2) cells were frozen at 5 × 10^6^ cells/ml in Cryostor^®^ CS10 (BioLife Solutions, StemCell Technologies, Canada) to constitute the MCB, consisting of 1 ml aliquots in 1.8 ml Nunc cryovials (Thermo Fisher Scientific). A controlled rate biological freezer was used (Biofreeze^®^ BV45, Consartic, Germany) and the vials were stored in liquid nitrogen gas phase.

A fraction of the cells were maintained in culture for a further four passages (up to P6), to constitute the PPCB, which was frozen (same conditions used for the MCB) for QC purposes.

### Large-Scale Manufacturing of GMP-Exo-CPC

For each manufacturing run, frozen CPC from MCB (P2) were thawed and cultured (8–10 × 10^4^ cells/cm^2^ initial seed) in StemMACS-MSC expansion Media kit XF or MSC-Brew GMP Medium, to obtain 16 CellBIND^®^Hyperflask^®^ culture vessels. At P4, the medium was removed, the culture vessels were washed twice with DPBS, then the production medium was added (about 500 ml/vessel). After 14 days at 37°C 5% CO_2_, the CM was collected (total 8 l) in sterile bottles (Corning).

The EPC harvested after CM collection were frozen (3.3 × 10^6^ cells/ml, same freezing medium used for the MCB) for QC purposes.

The Exo isolation was carried out in a closed system using the ÄKTA^TM^ Flux 6 instrument (previously sanitized according to the manufacturer’s instructions), through the following process phases:

(1) *CLARIFICATION:* the CM-containing bottles were connected to the instrument circuit for clarification through a ULTA Pure HC 0.6/0.2 μm Capsule Filter (GE Healthcare); the instrument transfer pump was used and the clarified CM was collected directly in the instrument tank (*process vessel*).
(2) *CONCENTRATION:* the activation of the instrument feed pump initiated the concentration by TFF. Instrument parameters (flow rate, transmembrane pressure) were set, according to manufacturer’s instructions, to minimize the “shear stress” in order to preserve Exo integrity. The permeate, containing components below the 300 kDa cut-off, was collected in a waste container, while the retentate (enriched in Exo) was recirculated to the *process vessel*. At 300–500 ml, the concentration was stopped.
(3) *DIAFILTRATION:* the concentrated CM contained in the *process vessel* was diluted in formulation buffer (Plasma-Lyte A^®^ solution, total five volumes in five diafiltration cycles), with the aim to obtain a replacement of the initial production medium greater than or equal to 95%. The diluted solution was concentrated through the same hollow fiber cartridge used for the previous phase, until reaching a 200–300 ml volume in the *process vessel*.
(4) *RECOVERY:* the solution contained in the *process vessel* and in the instrument circuit was harvested through the bottom sample port in a *post-process bag*.
(5) *STERILIZING FILTRATION:* the *post-process bag* was connected to the instrument circuit for sterilizing filtration through a Sterile Millipak^®^-20 Filter Unit 0.22 μm (Merck Millipore); the instrument transfer pump was used and the sterile product (275–350 ml) was collected directly in a *final product bag*.
(6) *FILLING:* the final product was filled in 0.5, 1, and 3 ml aliquots in 1.8 and 4.5 ml Nunc cryovials (Thermo Fisher Scientific), as appropriate. The vials were frozen and stored at -80°C, as GMP-Exo-CPC.

### CPC Count

Frozen aliquots of MCB, PPCB, and EPC were thawed and cell counting was performed with the EVE^TM^ Automated Cell Counter (NanoEnTek Inc., United States). The same system was used for in process controls (cell number and viability) during CPC culture.

### CPC Immunophenotype Analysis

Surface markers expressed on CPC were analyzed by flow cytometry using the MSC Phenotyping Kit human (Miltenyi Biotec GmbH) and MACSQuant Analyzer (Miltenyi Biotec GmbH).

### CPC Apoptosis Analysis

The BD Pharmingen^TM^ Annexin V FITC apoptosis detection kit (Becton Dickinson, United States) was used according to manufacturer’s instructions. In the flow cytometry plots, the following cell populations were identified: viable = annexin V negative/PI negative; early apoptotic = annexin V positive/PI negative; late apoptotic/necrotic = annexin V positive/PI positive plus annexin V negative/PI positive.

### CPC RT-PCR

The expression of transcription factors was evaluated by RT-PCR.

The genes *GATA4* (Gene ID: 2626), *TBX5* (Gene ID: 6910), *TBX18* (Gene ID: 9096), and *MESP1* (Gene ID: 55897) were selected according to literature data (Gallagher et al., 2014; Palpant et al., 2015) as CPC markers, representing mesodermal (TBX5, TBX18) and cardio-specific (GATA4, MESP1) transcription factors. As reverse transcription and amplification controls, primers on the *GAPDH* gene (Gene ID: 2597) were used.

### Nanoparticle Tracking Analysis

Exo-CPC particles were evaluated by NTA, with the instrument NanoSight LM10 (Malvern Instruments, United Kingdom).

### Measurement of Total Protein Content

The QuantiPro^TM^ BCA Assay kit (Sigma-Aldrich/Merck) was used for total protein quantification; the samples underwent three freeze/thaw cycles to break Exo structure immediately before running the assay.

### Measurement of TSG101 Content

The “Human TSG101 ELISA Kit (Sandwich ELISA) – LS-F8581” (LSBio LifeSpan Biosciences Inc., United States) was used for TSG101 content quantification. The samples underwent three freeze/thaw cycles to break Exo structure immediately before running the assay (Kim et al., 2005).

### Flow Cytometry Analysis of Exosome Surface Markers

To characterize Exo surface markers, the MACSPlex Exosome Kit (Miltenyi Biotec GmbH) was used following manufacturer’s instructions. Fluorescence was measured by the MACSQuant analyzer.

### Transmission Electron Microscopy (TEM) – Negative Staining

Sample aliquots were absorbed on glow-discharged carbon-coated formvar nickel grids and negatively stained with uranyl acetate. The grids were examined by Talos L 120C electron microscope at 120 kV.

### Protein Isolation and Western Blot Analysis

Total proteins were extracted by lyzing exosomes or cells in the presence of protease inhibitors. Proteins were separated on gel and transferred onto a PVDF membrane. Anti-Periostin (Santa Cruz, United States), anti-TSG101, anti-GRP94, and anti-GAPDH (Abcam, United Kingdom) were used as primary antibodies; IRDye^®^ 680RD or 800CW goat anti-mouse or goat anti-rabbit (LI-COR Biosciences, United States) as secondary antibodies. The infrared signal was detected using the Odyssey CLx Detection System (LI-COR Biosciences).

### Sterility Test

The sterility test (Microbiological Control for Cellular Products, Eu. Ph 2.6.27) was carried out on MCB, PPCB, EPC, and GMP-Exo-CPC using an automated microbial detection system (BACT/ALERT 3D, bioMérieux, France).

### Bacterial Endotoxins Test

The bacterial endotoxins were tested in the MCB, PPCB, EPC, and GMP-Exo-CPC samples according to Eu. Ph. 2.6.14, using the endosafe^®^-PTS^TM^ system (Charles River, United States).

### Exosome Functional Assays

The anti-apoptotic activity of Exo was tested by an *in vitro* apoptosis/viability assay developed in house, based on the use of CPC and staurosporine (Sigma-Aldrich/Merck) as apoptosis inducer. Exo were added at different concentrations; vehicle (Plasma-Lyte A^®^ solution) and/or Exo from normal human dermal fibroblasts (Exo-F) were used as controls. Cells were stained with calcein and PI (Thermo Fisher Scientific) and fluorescence was measured as representative for viability and apoptosis, respectively.

The pro-angiogenic activity of Exo at different concentrations was measured by the V2a Kit (Cellworks, United Kingdom) according to manufacturer’s instructions. Tubule formation was visualized by optical microscopy and total tube length was determined. A quantification of CD31 expression was also performed.

### Animal Experiments

Myocardial ischemia was induced in healthy male Wistar rats (250–300 g body weight) anesthetized with a cocktail of tiletamine and zolazepam (40 mg/kg given intraperitoneally; Zoletil100, Virbac, France), intubated, and ventilated. The left anterior descending artery was ligated near its origin with a 4-0 silk suture. Thirty minutes after permanent coronary artery occlusion, the peri-infarct myocardial region was injected at three different points with a total of 100 μl Plasma-Lyte A^®^ containing 1 × 10^11^ GMP-Exo-CPC particles (*n* = 10) or Plasma-Lyte A^®^ only (vehicle) as control (*n* = 5). The Exo concentration used was optimized based on previous dose–response studies.

In the sham-operated group (*n* = 5), the left coronary artery was not ligated. The chest was then closed, pneumothorax was reduced, and the rats were treated with meloxicam during postsurgical recovery. Sedated rats underwent transthoracic echocardiography at 7 and 28 days post-MI using the Vevo2100 echocardiography system (VisualSonic System) equipped with a 12-MHz linear transducer. Under ECG monitoring of heart rate, 2D images of the hearts were acquired in long-axis and short-axis. LVEF was measured with the biplane Simpson’s method; LVESV and LVEDV were also determined. Echocardiograms were evaluated by two independent examiners blind to the treatment protocol.

### Statistical Analysis

All the data were expressed as the mean ± SD.

Student’s *t*-test was used for the comparison of two groups. In case of multiple groups, one-way analysis of variance was performed, and the following pairwise comparisons were tested with the Student’s *t*-test, correcting the alpha according to the number of planned comparisons (Bonferroni correction). Statistical significance was accepted for *p*-values < 0.05 (^∗^), <0.01 (^∗∗^), or <0.001 (^∗∗∗^).

## Results

### Definition of Optimal GMP Grade Conditions for CPC Isolation and Culture

#### CPC Can Be Efficiently Isolated and Expanded in GMP Grade Xeno-Free Conditions

Three different CPC isolation and culture methods were compared, as summarized in **Table 1**.

**FIGURE 1:**
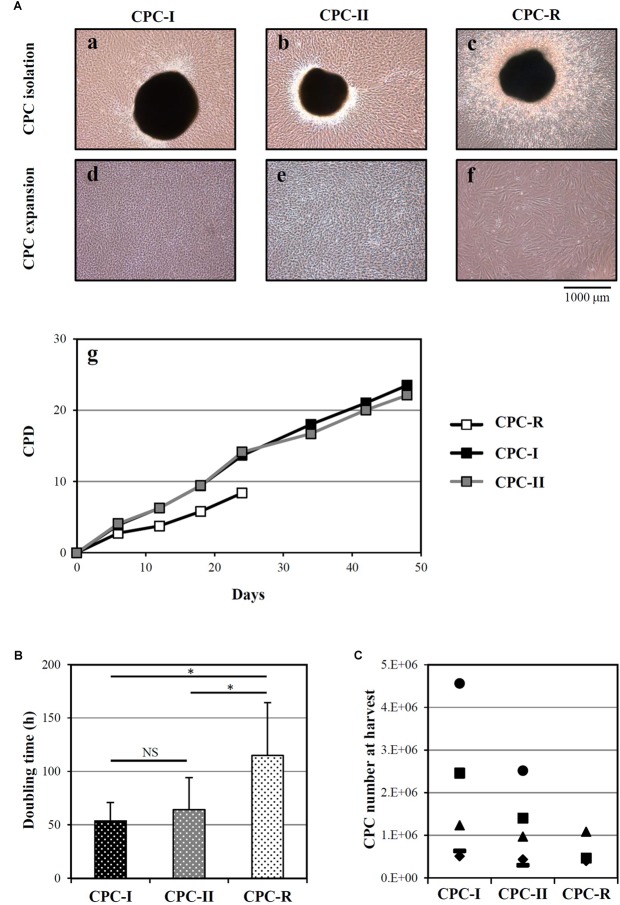
Isolation and culture of CPC. **(A)** Representative images of CPC outgrowth from cardiac fragments **(a–c)** and CPC expansion **(d–f)**, under the GMP grade methods I and II (CPC-I and -II) and the research grade method (CPC-R). The cumulative population doublings (CPD) over time is also shown **(g)** for the same representative experiment. **(B)** Doubling times (mean + SD, *n* = 5) of CPC cultured in the different conditions. NS, not significant; ^∗^*p* < 0.05, one-way ANOVA with Student’s *t*-test with Bonferroni correction. **(C)** CPC number at harvest from the outgrowth plates, by culture conditions. CPC-I and -II: *n* = 5; CPC-R: *n* = 3.

Cardiac progenitor cells were successfully isolated from heart tissue fragments in all the tested conditions, as shown in **Figure 1A** for a representative donor. The morphology of outgrowing GMP grade cells, both CPC-I and CPC-II (panels a and b) was similar to that of research grade cells CPC-R (panel c). During cell expansion, at confluence, CPC-I and CPC-II (panels d and e) appeared smaller and more actively replicating than CPC-R (panel f). The higher replication rate of GMP grade cells is indeed evidenced by the growth curves of CPC-I and CPC-II, when compared to that of CPC-R (panel g). Moreover, both CPC-I and CPC-II showed regular cell growth up to culture passage 8 (P8), whereas the CPC-R culture was stopped after five passages only, as the cells were no longer able to adhere properly to the culture flasks.

The higher growth rate of CPC-I and CPC-II in respect to CPC-R was confirmed analyzing parallel cultures derived from five different donors: **Figure 1B** shows that the mean doubling time was significantly lower for CPC-I (52 h) and CPC-II (61 h) than for CPC-R (105 h).

**Figure 1C** refers to five different donors, in which the initial heart tissue specimen was divided in two to three equal aliquots, each processed in a distinct condition. No difference was detected in the number of CPC harvested in different conditions.

Overall, these data indicated that both the “GMP grade I” and “GMP grade II” conditions were suitable for CPC isolation and expansion.

The “GMP grade I” condition was chosen for scale-up experiments based on empirical considerations: in the first step for CPC isolation (**Figure 9** and **Supplementary Figure S5**), the heart tissue fragments appeared to adhere more tightly to the plastic in “GMP grade I” than in “GMP grade II” condition, facilitating the manipulation process.

#### CPC Cultured in GMP Grade Conditions Stably Express MSC Markers as Well as Mesodermal- and Cardiac-Specific Transcription Factors

**FIGURE 2:**
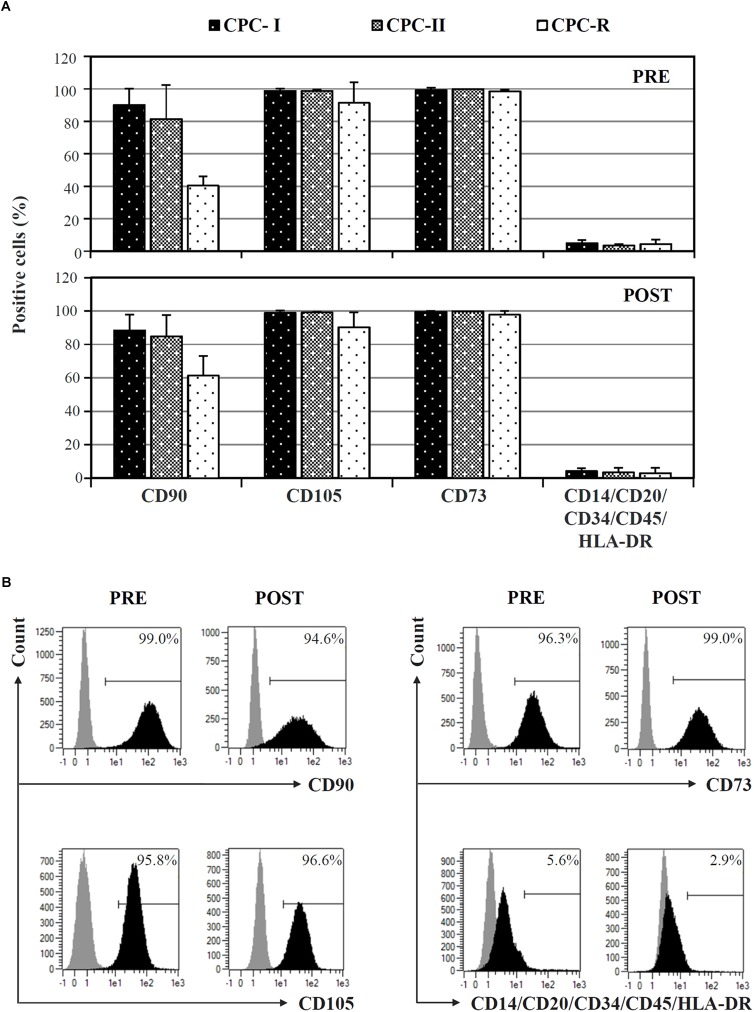
FACS analysis of CPC cultured in different conditions. **(A)** Comparison of surface markers expression on CPC cultured in GMP conditions (CPC-I and -II, *n* = 5) or research conditions (CPC-R, *n* = 3), before (PRE) and after (POST) 7-day *in vitro* starvation for Exo production. Bars: mean + SD. **(B)** Histogram plots of surface markers expression of CPC-I, before (PRE) and after (POST) starvation for Exo production. Gray peaks: staining with isotype control antibodies; black peaks: staining with antibodies specific for the reported markers.

Cardiac progenitor cells cultured in both research- and GMP-grade conditions expressed typical mesenchymal stem cell markers (CD73, CD90, CD105), and not hematopoietic markers, when analyzed before starvation for Exo release (**Figure 2A**, PRE). The expression was maintained during Exo production (**Figure 2A**, POST). Curiously, CD90 was less expressed and CD105 showed a higher variability in CPC-R than in CPC-I and CPC-II. A representative profile of surface markers expression on CPC-I, before (PRE) and after (POST) the starvation for exosome release, is shown in **Figure 2B**.

**FIGURE 3:**
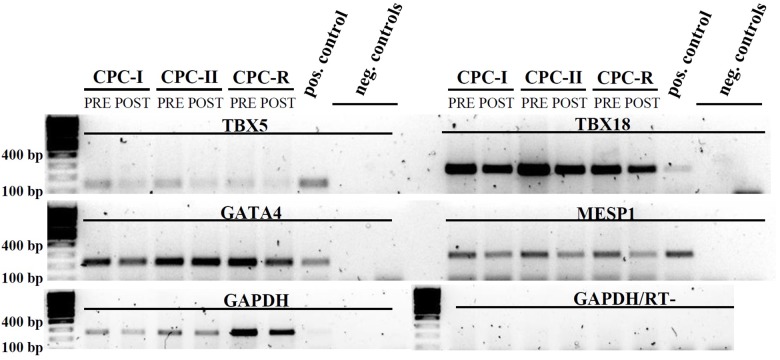
RT-PCR analysis of CPC cultured in different conditions. Mesodermal markers: *TBX5* and *TBX18*; cardiac markers: *GATA4* and *MESP1*. CPC samples were taken before (PRE) and after (POST) starvation for Exo production. Positive control: cardiac tissue. Amplification control: GAPDH. Negative controls (run in duplicate): reverse transcription negative sample with GAPDH primers. Gel images were cut above the primer dimers bands.

The same CPC were tested for the expression of mesodermal (TBX5, TBX18) and cardiac specific (GATA4, MESP1) transcription factors by RT-PCR: the expression was maintained in all the conditions, before (PRE) and after (POST) starvation for exosome production (**Figure 3**).

#### CPC Cultured in GMP Grade Conditions Release Exo

To evaluate the ability of CPC cultured in different conditions to release Exo, CM samples were concentrated and analyzed by NTA.

**FIGURE 4:**
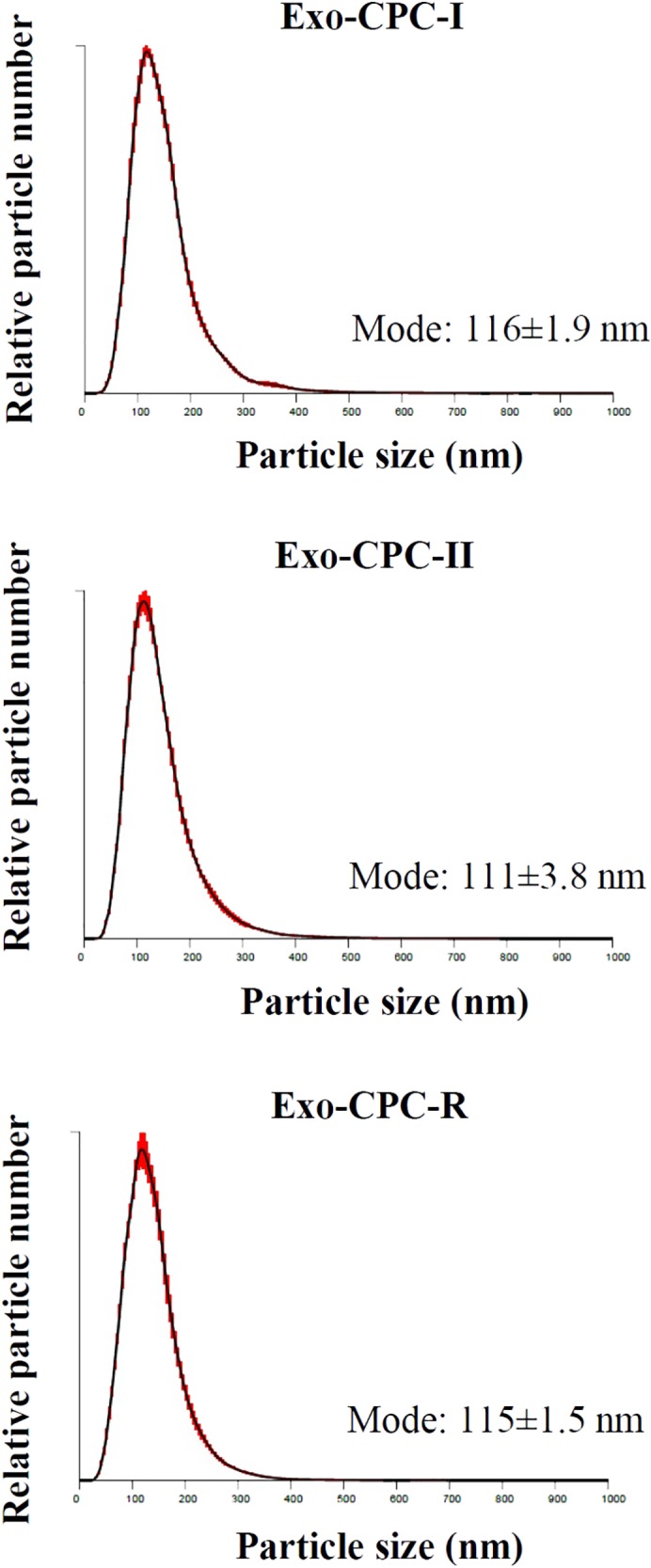
NTA profiles of Exo released by CPC cultured in different conditions. Size distribution graphs of the Exo from CPC expanded in GMP and research conditions. Red error bars indicate ±1 standard error of the mean. The mean ± standard error (*n* = 5) of the distribution modes from each replicate are shown.

As shown in **Figure 4**, all CPC were releasing particles showing the typical size distribution of Exo vesicles, with peaks in the 111–116 nm range, which fits with the 30–150 nm size range described in literature (Shedden et al., 2003; Cervio et al., 2015; Barile et al., 2017b; Sluijter et al., 2018).

No differences were observed in size distribution among the Exo produced by CPC cultured under the different conditions.

In western blot analysis (**Supplementary Figure S1**), the specific Exo marker TSG-101 and Periostin, a protein enriched in CPC-derived EV (Gouin et al., 2017), were detected in Exo preparations from CPC-I, CPC-II, and CPC-R, while the cell marker GRP94 was not present.

Flow cytometry analysis (**Supplementary Figure S2**) showed that Exo from CPC cultured in all the conditions expressed Exo markers (CD9, CD63, CD81) as well as CD105 and CD44, typical of CPC.

#### Exo Derived From CPC Cultured in GMP Grade Conditions Mediate Anti-apoptotic Activity

**FIGURE 5:**
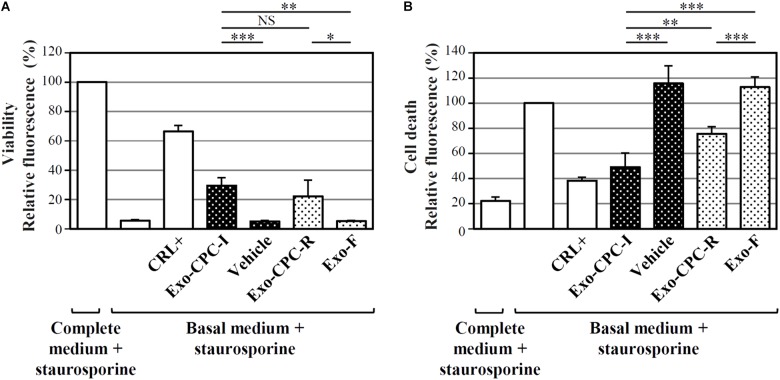
*In vitro* anti-apoptotic activity of Exo released by CPC cultured in different conditions. Apoptotic agent: 1 mM staurosporine. CRL + = 10% FBS; black dotted bars = Exo-CPC-I and control (vehicle = Plasma-Lyte A^®^); white dotted bars = Exo-CPC-R and control (Exo-F). Exo were added at 3 **×** 10^7^ particles/ml (100 μl/well), while the vehicle was added at the same volume as Exo-CPC-I. **(A)** Results from calcein staining. The treatment with complete medium + staurosporine is the 100% survival reference. **(B)** Results from PI staining. The treatment with the basal medium + staurosporine is the 100% cell death reference. Bars: mean + SD (*n* = 5). NS, not significant, ^∗^*p* < 0.05, ^∗∗^*p* < 0.01, ^∗∗∗^*p* < 0.001, one-way ANOVA with Student’s *t*-test with Bonferroni correction.

Exo produced by cells expanded in different conditions were analyzed for their functional activity, in terms of CPC protection from staurosporine-induced apoptosis (**Figure 5**). As shown in **Figure 5A**, cells cultured in the presence of Exo produced by both CPC-I (Exo-CPC-I) and CPC-R (Exo-CPC-R) showed higher level of viability (30 ± 5 and 22 ± 11%, respectively) compared to controls (vehicle, 5 ± 1%, and Exo-F, 5 ± 1%). Accordingly, cell death was reduced with both Exo-CPC-I and Exo-CPC-R (4 ± 11 and 76 ± 6%) in respect to vehicle (116 ± 14%) or Exo-F (113 ± 8%; **Figure 5B**).

The protective effect of Exo-CPC was also observed on HL-1 cardiomyocytic cells (**Supplementary Figure S3**).

### Exo-CPC: Upstream Process Optimization and Scale-Up

To optimize the Exo production phase (the upstream process), CPC expanded in culture up to P4 were starved in production medium for different times and in different scales, from 15 ml to 8 l.

As a quantitative indicator of Exo release in different conditions, the Exo-specific TSG101 protein was tested in CM extracts; total protein content was also determined as a measure of unspecific contamination.

**FIGURE 6:**
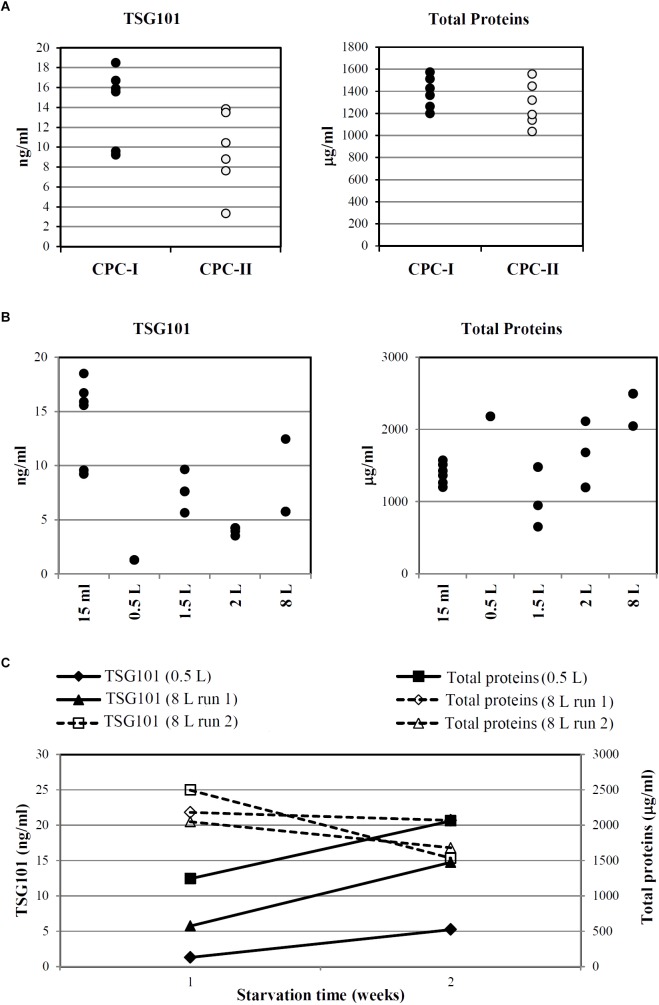
Optimization and scale-up of Exo-CPC production. **(A)** TSG101 content and total protein content in CM from CPC-I and -II. Production scale: 15 ml; cells starved for 7 days before medium collection. Each dot represents the analyte concentration in CM from a single culture. **(B)** TSG101 content and total protein content in CM from CPC-I on different culture volumes; cells starved for 7 days before medium collection. Each dot represents the analyte concentration in CM from a single culture. **(C)** Time course of TSG101 and total protein content in CM collected from CPC-I at 1 week (7 ± 1 days) and 2 weeks (14 ± 1 days) during starvation. Production scale: 0.5 and 8 l. Paired Student’s *t*-test at 2 weeks versus 1 week starvation: *p* < 0.05 for TSG101 content, not significant for total proteins content.

**Figure 6A** indicates that, despite intra-sample variations, TSG101 was consistently detected in CM harvested after 7 days of starvation (15 ml scale, *n* = 6) from both CPC-I and CPC-II.

As reported above, CPC-I condition was chosen for scale-up. **Figure 6B** shows that TSG101 was detected in CM harvested after 7 days of starvation in the whole scale range, from 15 ml to 8 l.

Time course experiments were performed with CPC-I in the 500 ml (*n* = 1) and 8 l (*n* = 2) scales (**Figure 6C**): the TSG101 content was significantly increased at 2 weeks, while the total protein content was stable.

After 1 as well as 2 weeks in production medium, CPC-I appeared healthy and well adherent (**Supplementary Figure S4A**), and they maintained their phenotypic characteristics (**Supplementary Figure S4B**). No relevant levels of apoptosis were detected (**Supplementary Figure S4C**).

CPC-I floating non-adherent cells were counted after 14 days starvation, and found to represent 5% of the total cells. These data indicate that the serum-free production medium guarantees the survival of the large majority of cells for up to 14 days, while maintaining their characteristics.

Based on these results, the starvation time for large-scale GMP-Exo-CPC production was set to 14 days.

### Exo-CPC: Downstream Process Optimization and Scale-Up

To proceed toward Exo-CPC isolation (the downstream process), the CM was first clarified in order to remove cell debris and aggregates, through centrifugation and/or 0.22 μm filtration (details in the section “Materials and Methods”).

Concentration through membranes with defined cut-off was then performed, with the aim of retaining Exo in the concentrate, while removing liquid and contaminants.

To follow Exo recovery throughout the process, the Exo-specific TSG101 protein was tested in extracts from “in process” samples collected at different points; total protein content was also determined, as impurity indicator.

**FIGURE 7:**
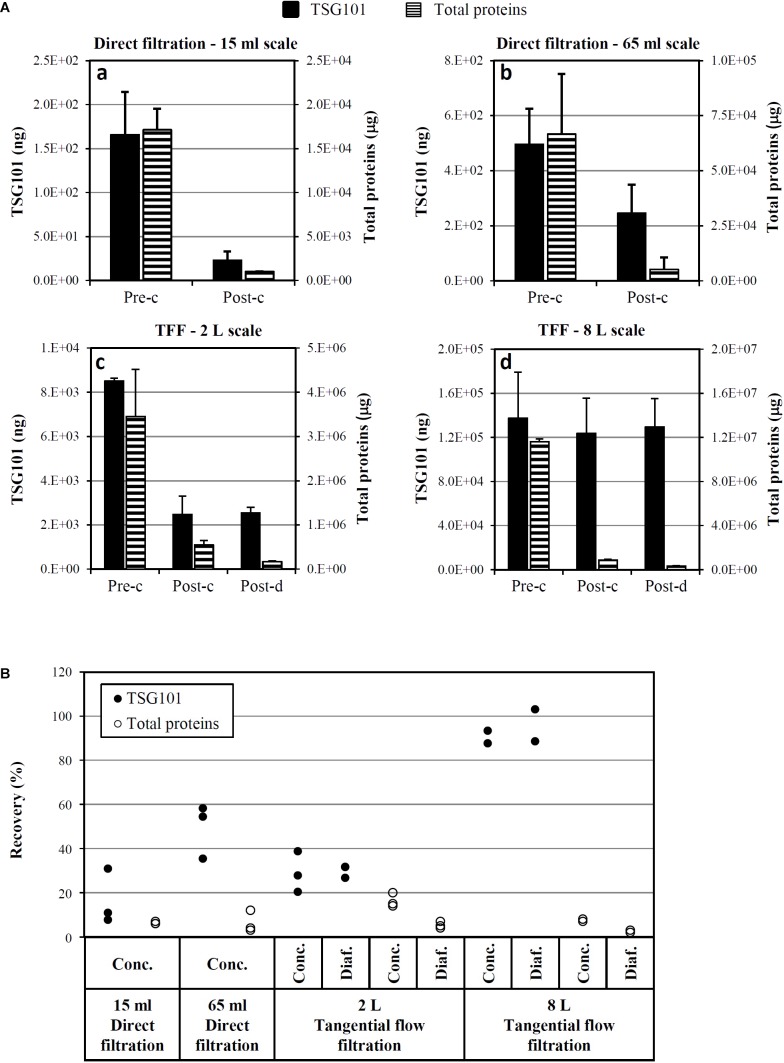
Optimization and scale-up of Exo-CPC isolation. **(A)** Quantitative analysis of TSG101 and total proteins before and after concentration **(a–d)** and diafiltration **(c,d)** by different techniques. Mean + SD with *n* = 3 (**a–c**; CM collected after 1 week starvation) or *n* = 2 (**d**, CM collected after 2 weeks starvation). **(B)** TSG101 and total protein recovery as a percentage of the quantity measured in the initial CM (pre-concentration). Each dot is the result of an independent experiment. Pre-c, before concentration; post-c, after concentration; post-d, after diafiltration.

As shown in **Figure 7A**, two different filtration systems were tested, in different scales, from 15 ml to 8 l of CM: direct filtration (scales: 15 and 65 ml, three experimental runs each, CM collected after 1 week starvation) and TFF (scales: 2 l, three runs, CM collected after 1 week starvation, and 8 l, two runs, CM collected after 2 weeks starvation). Up to about 500 ng of TSG101 were loaded on direct filtration (panels a and b), obtaining around 240 ng of TSG101 after concentration; up to about 140 μg of TSG101 were loaded on TFF (panels c and d), obtaining around 120 μg of TSG101 after concentration. Consistent total protein removal was observed in both systems.

In the TFF system, diafiltration was also performed after concentration, in order to formulate Exo-CPC in Plasma-Lyte A^®^, a solution suitable for *in vivo* administration in humans.

The yields of single process runs are shown in **Figure 7B**. The best performing process was 8 l TFF, which guaranteed a TSG101 yield of at least 89%, with a concomitant total protein removal of 97–98%.

Based on these results, TFF was selected as the method of choice for large-scale isolation of GMP-Exo-CPC.

### Generation of CPC Master Cell Bank and Post-production Cell Bank

Heart tissue specimen from a dedicated donor was processed exclusively for CPC banking in the selected GMP condition (“GMP grade I”).

At day 15 after the seeding of tissue fragments, about 4.6 × 10^6^ CPC were harvested and expanded for 36 days up to 1.4 × 10^8^ cells at P2, then frozen to constitute the MCB. A small fraction of the same cells (5 × 10^6^) were reseeded and expanded until culture passage 6 (P6), then frozen as PPCB. This last cell bank was generated for QC purposes only, in order to check the quality of the CPC maintained in culture well beyond P4, the passage when the production of Exo was planned. Regulatory guidelines (I.C.H., 2018) indeed require the analysis of PPCB (“cells at the limit of in vitro cell age used for production”), together with the MCB, to evaluate the stability of the cells in culture conditions.

Master cell bank and PPCB were then tested according to a defined QC plan. As explained more in detail in Section “Discussion,” the approval for the preclinical or clinical use is based on criteria (the specifications) defined for each QC test. The results of such exhaustive testing are shown in **Table 2**.

**Table 2:** Quality control of CPC master cell bank and post-production cell bank.

Parameter	Method	Specifications	Results	
			MCB	PPCB
Sterility	Microbiological control for cellular products	Absence of growth	Absence of growth	Absence of growth
Bacterial endotoxin (EU/ml)	Quantitative LAL test	<5.0	<5.0	<5.0
Mycoplasma	PCR	Negative	Negative	Negative
Cell concentration (cells/ml)	Trypan blue, automated cell counting	≥4 × 10^6^	4.48 × 10^6^	4.88 × 10^6^
Cell viability (%)		≥70	78	86
CD90 (% positive cells)	Flow cytometry	≥90.0	99.0	99.8
CD105 (% positive cells)		≥90.0	95.8	95.6
CD73 (% positive cells)		≥90.0	96.3	99.7
CD14/20/34/45-HLA DR (% positive cells)		<10.0	5.6	7.6
GATA4 expression (mRNA)	RT-PCR	Positive	Positive	Positive
TBX5 expression (mRNA)		Positive	Positive	Positive
TBX18 expression (mRNA)		Positive	Positive	Positive
MESP1 expression (mRNA)		Positive	Positive	Positive

All the results met the specifications: both MCB and PPCB were sterile, negative for bacterial endotoxins and mycoplasma (the safety tests). They had good viability (the potency test) and they were positive for mesenchymal markers, negative for hematopoietic surface markers, and expressed the mesodermal and cardiac-specific transcription factors (the identity tests).

On this basis, the MCB was approved for the large-scale production of GMP-Exo-CPC to be used in preclinical studies.

### Large-Scale Manufacturing of GMP-Exo-CPC

**FIGURE 8:**
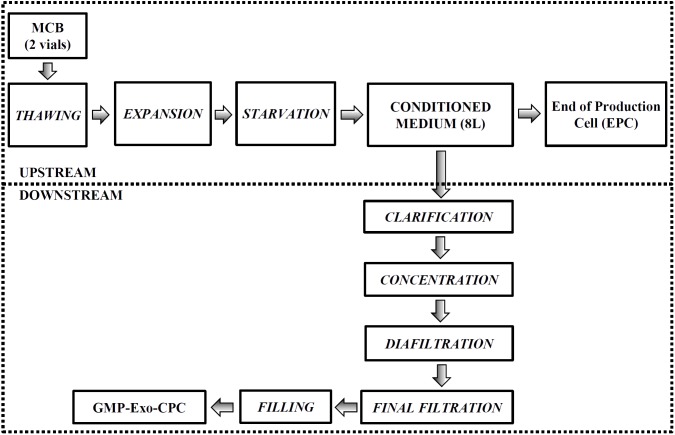
Large-scale GMP-Exo-CPC production chart. The upstream and downstream processes are grouped by the dotted boxes. See Section “Materials and Methods” for details.

The whole process is summarized in **Figure 8**. The results of two full scale production runs are shown here below.

#### Upstream Process: CM Production

Two full scale runs were conducted. For each run, two MCB aliquots were thawed (about 1 × 10^7^ cells) and expanded *in vitro* up to P4 (total cell culture surface > 2.5 m^2^). About 1 × 10^8^ cells (first run: 0.9 × 10^8^, second run: 1.2 × 10^8^) were seeded, expanded in culture medium until confluence, then induced to release Exo by starvation in production medium for 2 weeks. About 8 l of Exo-containing CM were collected, containing 20.6 (first run) and 16.1 (second run) ng/ml TSG101.

After CM collection, cells were harvested (first run: 5.9 × 10^8^, second run: 7.2 × 10^8^ cells) and part of them (9 × 10^7^ corresponding to 1/6–1/8 of the total cell number) were frozen as EPC.

#### Downstream Process: GMP-Exo-CPC Isolation

Exo were isolated as summarized in **Figure 8** and detailed in Section “Materials and Methods.”

The two full scale runs yielded 2.9 × 10^13^ and 3.1 × 10^13^ exosome particles (corresponding to 100 μg and 75 μg of TSG101, respectively), in final formulation buffer.

The final solutions containing the Exo were aliquoted in cryovials and frozen at -80°C, to constitute GMP-Exo-CPC Lot 1 and Lot 2, respectively.

### GMP-Exo-CPC Quality Control

The results of the exhaustive QC testing of GMP-Exo-CPC are shown in the first section of **Table 3**.

**Table 3:** Quality control of GMP-Exo-CPC lots and EPC.

GMP-Exo-CPC				
**Parameter**	**Method**	**Specifications**	**Results**	
			**GMP-Exo-CPC lot 1**	**GMP-Exo-CPC lot 2**
Sterility	Microbiological control for cellular products	Absence of growth	Absence of growth	Absence of growth
Bacterial endotoxin (EU/ml)	Quantitative LAL test	<5.0	<5.0	<5.0
TSG101 (ng/ml)	ELISA	FIO	285	272
Total proteins (μg/ml)	BCA assay	FIO	1008	840
Particle concentration (p/ml)	Nanoparticle tracking analysis	FIO	8.26 × 10^10^	1.12 × 10^11^
Particle size (nm)		50–150	121	143
CD9 expression	Flow cytometry	Positive	Positive^#^	Positive^#^
CD63 expression		Positive	Positive^#^	Positive^#^
CD81 expression		Positive	Positive^#^	Positive^#^

**EPC**				

**Parameter**	**Method**	**Specifications**	**Results**	
			**EPC lot 1**	**EPC lot 2**

Sterility	Microbiological control for cellular products	Absence of growth	Absence of growth	Absence of growth
Bacterial Endotoxin (EU/ml)	Quantitative LAL test	<5.0	<5.0	<5.0
Mycoplasma	PCR	Negative	Negative	Negative
Cell concentration (cells/ml)	Trypan blue, automated cell counting	>2 × 10^6^	2.24 × 10^6^	2.37 × 10^6^
Cell viability (%)		≥70	81	87
CD90 (% positive cells)	Flow cytometry	≥90.0	97.3	94.6
CD105 (% positive cells)		≥90.0	98.8	96.6
CD73 (% positive cells)		≥90.0	99.7	99.01
CD14/20/34/45-DR (% positive cells)		<10.0	6.7	2.87
GATA4 expression (mRNA)	RT-PCR	Positive	Positive	Positive
TBX5 expression (mRNA)		Positive	Positive	Positive
TBX18 expression (mRNA)		Positive	Positive	Positive
MESP1 expression (mRNA)		Positive	Positive	Positive

**FIGURE 9:**
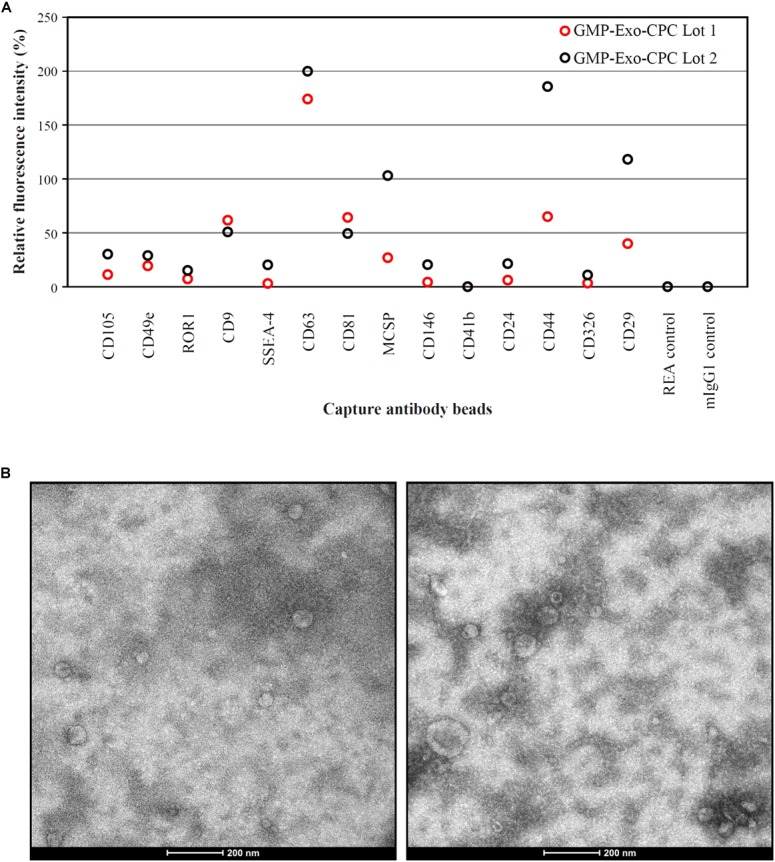
Characterization of GMP-Exo-CPC. **(A)** Flow cytometry analysis. Two lots of GMP-Exo-CPC were analyzed with the MACSPlex Exosome Kit. Data are represented as APC mean fluorescence intensity normalized to the mean signal intensity obtained with the anti-CD9, anti-CD63, and anti-CD81 beads. **(B)** Representative TEM images.

*FIO: for information only; ^#^see **Figure 9**.*

All the results met the specifications: both lots were sterile and negative for bacterial endotoxins (safety tests). Moreover, they contained Exo of the expected size expressing the CD9, CD63, and CD81 surface markers (identity/purity tests).

Extended surface marker characterization (**Figure 9A**) showed that the GMP-Exo-CPC also expressed CD105 and CD44, antigens typical of CPC, as well as CD146, that was already detected on Exo-CPC-R (Barile et al., 2014).

A number of other markers were not expressed, including HLA and hematopoietic antigens. The full list of markers evaluated is reported in **Supplementary Table S1**.

GMP-Exo-CPC were also analyzed by TEM, and representative images are shown in **Figure 9B**. Western blot analysis (**Supplementary Figure S1**) was performed to detect the specific Exo marker TSG-101, Periostin, a protein enriched in CPC-derived EV (Gouin et al., 2017), as well as the cell marker GRP94, used as an indicator of cell contaminants from cell culture. TSG101 was consistently detected in Exo preparations and cell lysate. Periostin was strongly enriched in Exo and was not detectable in cell lysate. GRP94 was consistently detected in CPC and poorly, if not, present onto Exo, suggesting lack of substantial contamination by cell debris.

The GMP-Exo-CPC lots were also tested for TSG101 content (a further identity test), total protein content (purity test), and particle concentration.

Moreover, *in vitro* and *in vivo* functional activities were assessed as described below (potency test).

#### GMP-Exo-CPC Mediate Anti-apoptotic Activity *in vitro*

To test the anti-apoptotic activity of GMP-Exo-CPC, the same apoptosis/viability assay above described was used.

**FIGURE 10:**
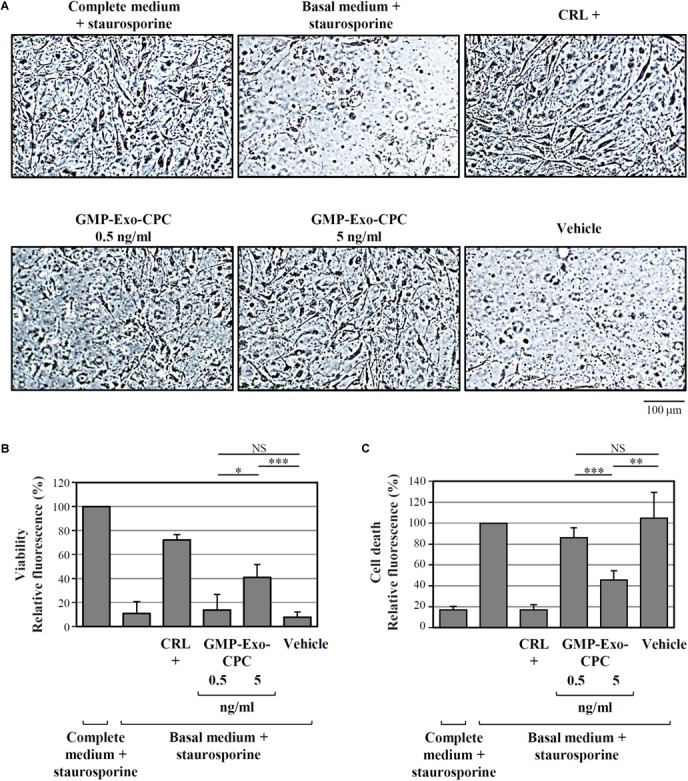
*In vitro* anti-apoptotic activity of GMP-Exo-CPC. Apoptotic agent: 1 mM staurosporine. CRL + = 10% FBS; GMP-Exo-CPC doses: 0.5 and 5 ng of TSG101/ml (100 μl/well); vehicle = Plasma-Lyte A^®^ added at the same volume as GMP-Exo-CPC. **(A)** Representative images at 10x magnification of the cells after the indicated treatments. **(B)** Results from calcein staining. The treatment with the complete medium + staurosporine is the 100% survival reference. **(C)** Results from PI staining. The treatment with the basal medium + staurosporine is the 100% cell death reference. Bars: mean + SD (*n* = 5). NS, not significant, ^∗^*p* < 0.05, ^∗∗^*p* < 0.01, ^∗∗∗^*p* < 0.001, one-way ANOVA with Student’s *t*-test with Bonferroni correction.

As depicted by the images from representative wells (**Figure 10A**) and by calcein AM staining results (**Figure 10B**), cells treated with Exo at the 5 ng/ml dose showed higher viability than cells treated with vehicle alone (GMP-Exo-CPC 0.5 ng/ml: 14 ± 13%, GMP-Exo-CPC 5 ng/ml: 41 ± 11%, vehicle 8 ± 4%).

In agreement with calcein experiment data, the addition of Exo protected CPC from cell death, as shown in **Figure 10C** by the PI staining results (GMP-Exo-CPC 0.5 ng/ml: 86 ± 10%, GMP-Exo-CPC 5 ng/ml: 46 ± 9%, vehicle 105 ± 25%).

In both calcein AM and PI readouts, the protective effect of Exo was dose-dependent and, at the highest dose, statistically significant if compared to the vehicle.

#### GMP-Exo-CPC Mediate Pro-angiogenic Activity *in vitro*

The pro-angiogenic activity of GMP-Exo-CPC was tested by measuring the length of tubules and the expression of the CD31 marker induced in human umbilical endothelial cells.

**FIGURE 11:**
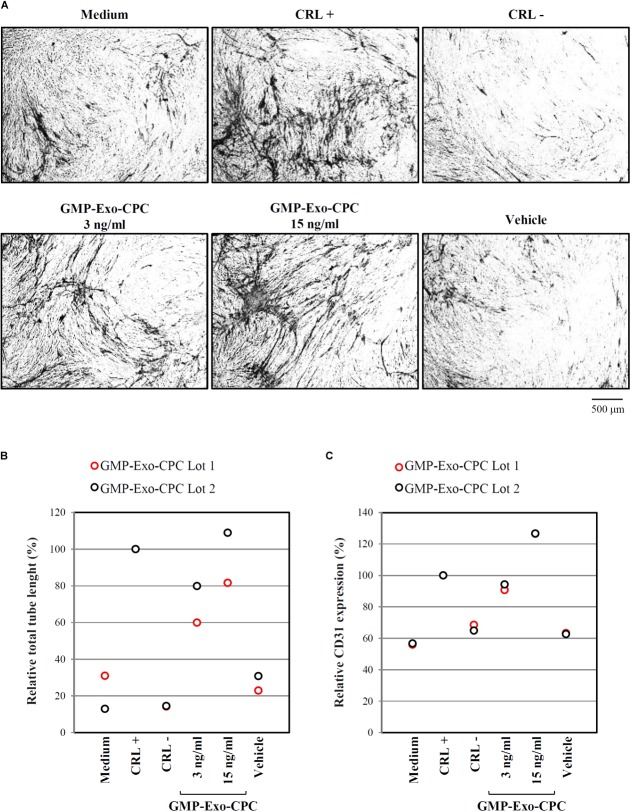
*In vitro* pro-angiogenic activity of GMP-Exo-CPC. GMP-Exo-CPC doses: 3 and 15 ng of TSG101/ml (0.5 ml/well). CRL + = VEGF; CRL – = suramin; vehicle = Plasma-Lyte A. **(A)** Representative images at 4x magnification of cells after the indicated treatments. CD31-stained tubules are visible in black. **(B)** Total tubule length detected by the AngioSys 2.0 Image Analysis Software. The mean length in VEGF treated cells was set to 100%. **(C)** CD31 expression detected by ELISA. The expression in VEGF treated cells was set to 100%.

Pictures of CD31-stained cells in different conditions are shown in **Figure 11A** and total tube lengths in **Figure 11B** (GMP-Exo-CPC 3 ng/ml: 60% for GMP-Exo-CPC Lot 1 and 80% for GMP-Exo-CPC Lot 2, GMP-Exo-CPC 15 ng/ml: 82% for GMP-Exo-CPC Lot 1 and 109% for GMP-Exo-CPC Lot 2, vehicle: 23 and 31%).

CD31 expression was higher in cells treated with Exo than in in cells exposed to vehicle (**Figure 11C**; GMP-Exo-CPC 3 ng/ml: 91% for GMP-Exo-CPC Lot 1 and 94% for GMP-Exo-CPC Lot 2, GMP-Exo-CPC 15 ng/ml: 127% for GMP-Exo-CPC Lot 1 and 127% for GMP-Exo-CPC Lot 2, vehicle: 63 and 63%). No statistical analysis was possible due to the low number of experiments.

#### GMP-Exo-CPC Show Therapeutic Efficacy in a Rat Preclinical Model

**FIGURE 12:**
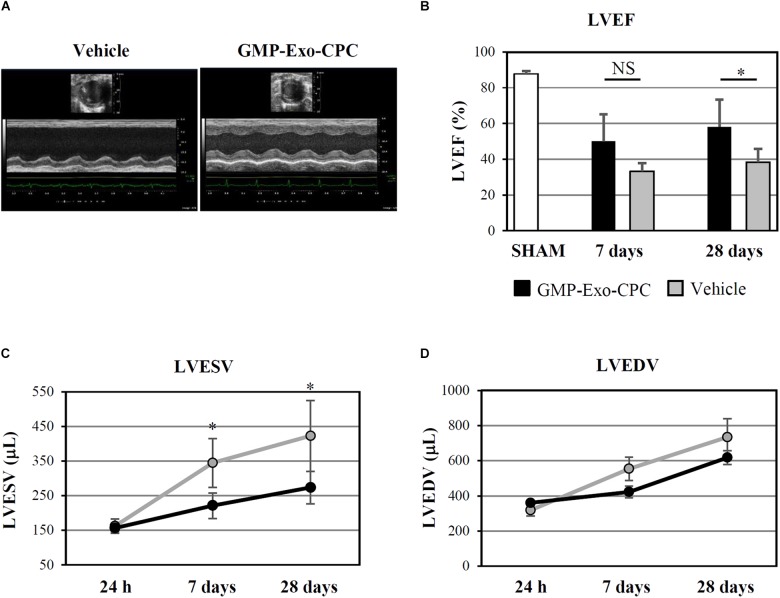
*In vivo* therapeutic effect of GMP-Exo-CPC. **(A)** Representative echocardiographic M-mode images of hearts injected with vehicle and GMP-Exo-CPC, taken at day 28 after MI. **(B)** LVEF in sham-operated rats at day 28 (SHAM, *n* = 5), in GMP-Exo-CPC injected rats (black bars, *n* = 10), and vehicle injected rats (gray bars, *n* = 5) 7 and 28 days after MI. Bars: mean + SD. Measurements of LVESV **(C)** and LVEDV **(D)** in GMP-Exo-CPC injected rats (black line) and vehicle injected rats (gray line) 24 h, 7 and 28 days after MI. Mean ± SD is shown. Vehicle = PlasmaLyte A^®^. ^∗^*p* < 0.05, Student’s *t*-test.

The functional activity of GMP-Exo-CPC was confirmed *in vivo*, in a rat model of permanent coronary artery occlusion (**Figure 12**).

GMP-Exo-CPC significantly improved LVEF after 4 weeks, whereas the vehicle alone was not effective (**Figure 12B**).

Moreover, GMP-Exo-CPC treatment was associated with a significantly lower increase of LVESV compared with vehicle at 1 as well as 4 weeks (**Figure 12C**), while LVEDV was not affected (**Figure 12D**).

### EPC Quality Control

As the GMP-Exo-CPC lots, the corresponding EPC underwent various QC tests, reported in the second section of **Table 3**.

All the results met the specifications: cells collected after both production runs were sterile, negative for bacterial endotoxins and mycoplasma, viable, positive for mesenchymal and negative for hematopoietic surface markers, as well as positive for mesodermal and cardiac-specific transcription factors.

## Discussion

### Exo as Therapeutic Tools

Exo from different cell types are being tested in clinical trials in several therapeutic areas, including cancer, diabetes, and wound healing (Lener et al., 2015; Barile and Vassalli, 2017; Sluijter et al., 2018). The safety and feasibility of the approach has been demonstrated in phase I–II immunotherapy studies for colon rectal cancer, melanoma, and non-small cell lung cancers using Exo from dendritic cells pulsed with tumor antigens; no relevant adverse events occurred and partial clinical responses were detected in some patients (Pitt et al., 2016).

Based on these encouraging results, Exo-based therapies may well be considered for other applications, including cardiovascular diseases (Barile and Vassalli, 2017; Barile et al., 2017b; Xu et al., 2017; Sluijter et al., 2018).

Exo released by CPC (Barile et al., 2012) have already shown a clear therapeutic potential: they inhibit cardiomyocyte apoptosis and promote angiogenesis *in vitro*, as well as reduce tissue damage, inhibit cardiomyocyte apoptosis, and promote angiogenesis *in vivo*, improving post-infarction cardiac function in a preclinical rat model of AMI (Barile et al., 2014).

Similarly, others have shown the beneficial effects of Exo released by CDC in AMI preclinical models in rats (Ibrahim et al., 2014) and pigs (Gallet et al., 2017).

CDC are considered cardiac progenitors derived from, but not identical, to CPC (Barile et al., 2017a). CDC by themselves have shown safety and potential efficacy in autologous cell therapy in humans, in a phase-I AMI clinical study (Makkar et al., 2012; Malliaras et al., 2014); they are now being tested in allogeneic contexts, in phase I-II trials for AMI (ClinicalTrials.gov Identifier: NCT01458405), dilated cardiomyopathy (ClinicalTrials.gov Identifier: NCT02293603), and Duchenne muscular dystrophy cardiomyopathy (ClinicalTrials.gov Identifier: NCT02485938). As Exo were identified as key mediators for the regenerative effects of CDC, trials with CDC-derived Exo are currently being planned (Gallet et al., 2017).

### Exo-CPC as a New Medicinal Product

To move toward clinical application in humans, Exo-CPC need to be developed as a novel medicinal product, according to the pertinent regulatory guidelines. Like other EV-based products, Exo should be considered as biological medicines, falling into the category of biopharmaceuticals as defined in EU, United States, and Australia (Lener et al., 2015; I.C.H., 2018). However, at variance with other biopharmaceuticals such as recombinant proteins or antibodies, EV are complex entities and may have much in common with their cell source. For this reason, the development of EV-based therapeutics should take into consideration not only the regulations for biologicals, but also those for cell based products and ATMP as defined in Lener et al. (2015).

The aim of the present work was indeed the development of production and QC methods for the large-scale preparation of Exo-CPC as a medicinal product, in accordance with current GMP. The focus has been set on both the cell source (CPC) and the product itself (Exo-CPC).

The starting point for development activities were the research grade methods used so far for isolation and culture of CPC and for small-scale production of Exo-CPC for pilot preclinical studies (Barile et al., 2014, 2018). Our approach has been focused on overcoming the main limitations of such methods: use of open culture vessels, poorly standardized reagents containing animal-derived components, hardly scalable steps.

On the analytical side, research grade CPC and Exo-CPC were not tested for safety. Sterility, endotoxin and mycoplasma assays were included in the QC panel for CPC cell banks and GMP-Exo-CPC, in order to approve them for preclinical studies in large animals. A more extensive safety panel, including adventitious viruses, karyotype, cell senescence and tumorigenicity, will be applied to next lots for first-in-human trials.

The *in vitro* efficacy (potency) of research grade Exo-CPC was tested by assays for anti-apoptotic and pro-angiogenic activity (Barile et al., 2014, 2018). In order to achieve the reproducibility and robustness required for their application in a GMP context, they have been revisited and optimized throughout the present work.

Objective of this work was also the establishment of a complete QC strategy suitable to obtain clinical trial authorization by the regulatory authority.

The approval of MCB as well as of GMP-Exo-CPC lots (release for clinical use) is indeed based on passing a defined list of tests. Each test result should fall within limits (specifications) defined at compendial level for parameters such as endotoxin, mycoplasma, and cell viability (European Pharmacopoeia, 2017), or set during the development phase for other product characteristics, on the basis of literature data and previous experimental results.

For example, the specifications for cell concentration for MCB, PPCB (**Table 2**) and EPC (**Table 3**) have been defined taking into consideration the concentration at which the cells had been frozen. For the MCB and PPCB the freezing concentration was 5 × 10^6^ cells/ml (see section “Materials and Methods”), thus the specification for cell concentration after thawing was set at 4 × 10^6^/ml, assuming a cell recovery of at least 80%, based on previous lab results. For the EPC, the freezing concentration was 3.3 × 10^6^ cells/ml, thus the specification for cell concentration after thawing was set at 2 × 10^6^/ml, assuming a cell recovery of at least 60%, based on previous lab results.

For some analytical parameters described here, the specification could not be defined yet, and the results will be collected FIO. This approach is in agreement with regulatory guidelines (I.C.H., 2018) for early phase (I-II) clinical trials; for advanced clinical phases, the specifications will be defined on the basis of such FIO results.

### Cell Source: CPC Derivation, Culture, and Banking Strategy

In the research context, open culture vessels were used during the initial tissue processing and for the adhesion of tissue fragments until the harvest of outgrowing CPC; bovine gelatin was used for the coating of culture vessels, FBS as supplement for culture medium, and porcine trypsin as cell detachment reagent.

During development, special flasks with reclosable lids and filter caps were introduced instead of dishes for the adhesion of tissue fragments, to minimize contamination risks since the beginning of the CPC growth.

Regarding the animal-derived reagents, regulatory authorities recommend any possible effort to remove them from ATMP processes, due to safety concerns. Moreover, it is well known that FBS could contain bovine EV, which may contaminate Exo-CPC preparations.

Several commercial serum-free culture media were tested as potential substitutes for the FBS-containing medium; among them, StemMACS-MSC expansion Media kit XF (Miltenyi Biotec GmbH) was selected based on its ability to stably support CPC growth, while maintaining cell characteristics. This is a standardized, complete, xeno-free medium designed for mesenchymal stem cells, that proved to be optimal for CPC; of note, its GMP version is now available, certified for *ex-vivo* therapeutic cell culture.

CELLstart^TM^ CTS^TM^, containing only components of human origin, and the fully synthetic Synthemax^®^ II-SC were chosen as alternative adhesion substrates and tested for gelatin replacement; among them, CELLstart^TM^ CTS^TM^ was eventually preferred because of its slightly better effect on CPC yield and Exo production. This reagent too is certified for *ex vivo* therapeutic cell culture.

Trypsin was replaced by the properly certified TrypLE^TM^ Select, a recombinant dissociation enzyme.

In summary, all the selected reagents are of high quality and properly certified, in line with regulatory expectations for ATMP and related products (Solomon et al., 2016).

The newly defined culture conditions allowed large-scale CPC expansion, up to 8 × 10^8^ cells. A cell bank was generated and approved for the production of GMP-Exo-CPC for preclinical studies. A new MCB will be produced in the GMP certified and classified area; it will be tested even more extensively to be approved for the manufacturing of GMP-Exo-CPC for first-in-man studies.

Additional tests will include *in vitro* and *in vivo* adventitious viruses, culture assay for mycoplasma, tumorigenicity, cell senescence, and karyotype.

### Medicinal Product: GMP-Exo-CPC Manufacturing

An essential issue for the future clinical application of Exo-CPC is the possibility to produce and isolate Exo in large amounts through a GMP compliant process.

So far, Exo for clinical applications were isolated by polymer-based precipitation, ultracentrifugation, TFF, density gradient or a combination of these methods, often applied as separate and open manufacturing passages (Lener et al., 2015; Konoshenko et al., 2018). Some of these technologies may favor the concomitant concentration of impurities (e.g., precipitation, ultracentrifugation) or may be hardly scalable (e.g., ultracentrifugation, density gradient; Xu et al., 2016). One of the first, well-described GMP methods (Lamparski et al., 2002) was developed for the production of dendritic cell-derived clinical grade Exo; this protocol, based on TFF followed by ultracentrifugation on sucrose density gradient, was successfully applied on 1–4 l of CM, resulting in 35–65% Exo yield.

Our method for GMP-Exo-CPC manufacturing has been implemented for 8 l of CM, but may be further scaled up, using bioreactors for CPC culture and CM production, as well as industrial TFF systems for Exo isolation. The absence of any ultracentrifugation step simplifies the process, allowing high final product yield (≥58%) with concomitant consistent reduction of contaminants (total protein removal 97–98%).

Moreover, efforts were made to develop an integrated closed circuit, encompassing the full downstream process (clarification, concentration, diafiltration, and final sterilizing filtration) in a single work station, the ÄKTA^TM^ flux 6 system (GE Healthcare).

The resulting GMP-Exo-CPC product contained about 3 × 10^13^ Exo particles, formulated in a clinical grade solution (Plasma-Lyte A^®^).

Such GMP-Exo-CPC were extensively characterized, according to a well-defined QC strategy, including compendial methods described in European Pharmacopoeia (2017), as well as non-compendial product-specific methods developed or optimized in house (manuscript in preparation) to achieve suitable sensitivity, precision, accuracy, reproducibility, and robustness, according to regulatory guidelines (I.C.H., 2018).

Among several commercially available ELISA kits for exosome proteins, the TSG101 ELISA used in this study was selected for its sensitivity, allowing for the detection of even the lowest Exo concentrations present in CM samples.

For purity evaluation, total protein content was determined, assuming that most of the proteins contained in CM could be soluble proteins, while a very small fraction of them could be Exo components. In the GMP-Exo-CPC lots described here, 1 mg of proteins corresponded to 1.08 × 10^11^ ± 3.63 × 10^10^ particles. A product with similar purity (2.2 × 10^11^ particles/mg of proteins) was successfully used by others in a large animal preclinical study (Gallet et al., 2017): Exo from CDC, injected directly into the heart muscle, were shown to reduce scar area and improve LVEF in porcine acute and chronic MI.

The potency of our GMP-Exo-CPC was demonstrated *in vitro* analyzing their anti-apoptotic and pro-angiogenic activity and confirmed *in vivo* in a rat model of permanent coronary artery occlusion, where GMP-Exo-CPC significantly improved cardiac function.

The *in vitro* potency assays were indeed designed with the aim of predicting the *in vivo* activity in a repeatable and robust fashion. In particular, the viability/apoptosis assay was developed starting from the corresponding research grade assay (Barile et al., 2014, 2018), optimizing test parameters (cell type, culture conditions, timing), and using a different and more quantitative detection method (fluorescence detection by plate reader instead of fluorescence microscopy followed by image analysis). The optimized test resulted more reproducible and suitable for formal GMP validation.

GMP-Exo-CPC were frozen at -80°C in single-dose aliquots, as convenient for a ready-to-use therapeutic product (Witwer et al., 2013). A stability study is ongoing; partial results obtained so far indicate no loss of functional activity in the storage conditions after 7 months.

A preclinical study in swine myocardial infarction model is now ongoing, to further test the safety and efficacy of the product.

The production of GMP-Exo-CPC lots to be used for first-in-man study will be carried out, starting from the MCB produced for clinical use, in a GMP certified and classified area. Each lot will be analyzed according to an extended QC panel in order to be released for clinical application.

## Conclusion

The large-scale process described here, based on an innovative approach combining several steps in an integrated circuit, led to a safe and well characterized GMP-Exo-CPC product. This manufacturing method may be also applied to Exo from other cell sources (e.g., mesenchymal stem cells, muscle cells, fibroblasts, nervous system cells, dendritic cells, etc.), for different clinical applications.

## Ethics Statement

Human tissue collection was carried out in accordance with the Helsinki Declaration and specific authorization by the local Ethics Committee (CE 2923). All subjects gave written informed consent in accordance with the Declaration of Helsinki. The animal study was carried out in accordance with the recommendations of Directive 2010/63/EU of the European Parliament and Animal Care Committee of Canton Ticino, Switzerland. The protocol was approved by the Animal Care Committee of Canton Ticino, Switzerland (TI-10-17).

## Author Contributions

GA, MR, LT, and LB conceived and designed the study. TT provided study material. GA, MR, and VL carried out the experimental work for process development and large-scale productions. EP, AB, LT, and SS were responsible for analytical development and quality control testing. GM, VB, and LB performed the *in vivo* experiment. GA, EP, AB, LB, and MR collected and analyzed the data. GA, EP, AB, and MR drafted the manuscript. LT, GV, LB, and MR critically revised it. All authors read and approved the final manuscript.

## Conflict of Interest Statement

Some of the authors (GA, EP, AB, VL, SS, LB, GV, LT, and MR) are among the inventors of the following patent applications (priority date 30/03/2017), issued by Foundation for Cardiological Research and Education:

• IT-102017000035315 DISPOSITIVO E METODO DI PRODUZIONE E PURIFICAZIONE DI ESOSOMI
• CH-00424/17 DISPOSITIVO E METODO DI PRODUZIONE E PURIFICAZIONE DI ESOSOMI
• PCT/EP2017/084747 DEVICE AND METHOD FOR PRODUCING AND PURIFYING EXOSOMES

The remaining authors declare that the research was conducted in the absence of any commercial or financial relationships that could be construed as a potential conflict of interest. The reviewer MB and handling Editor declared their shared affiliation.

## Abbreviations

AMI: acute myocardial infarction
ATMP: advanced therapy medicinal products
CDC: cardiosphere-derived cells
CM: conditioned medium
CPC: cardiac progenitor cells
CPC-I: cardiac progenitor cells grown under the GMP grade condition I
CPC-II: cardiac progenitor cells grown under the GMP grade condition II
CPC-R: cardiac progenitor cells grown under the research grade condition
DMEM: Dulbecco’s Modified Eagle Medium
DPBS: Dulbecco’s phosphate buffered saline
EPC: end of production cells
EV: extracellular vesicles
Exo: exosomes
Exo-CPC: exosomes from cardiac progenitor cells
Exo-CPC-I: exosomes released by CPC-I
Exo-CPC-II: exosomes released by CPC-II
Exo-F: exosomes from normal human dermal fibroblasts
Exo-CPC-R: exosomes released by CPC-R
FBS: fetal bovine serum
FIO: for information only
GMP: good manufacturing practices
GMP-EXO-CPC: Exo-CPC as a medicinal product produced according GMP regulation
LVEDV: left ventricular end diastolic volume
LVEF: left ventricular ejection fraction
LVESV: left ventricular end systolic volume
MCB: master cell bank
MI: myocardial ischemia
NTA: nanoparticle tracking analysis
PI: propidium iodide
PPCB: post-production cell bank
P2, P4, P6, P8: culture passage 2, 4, 6 or 8
QC: quality control
RT: room temperature
SD: standard deviation
TEM: transmission electron microscopy
TFF: tangential flow filtration
