# Supplementary material for: Exosomes From Human Cardiac Progenitor Cells for Therapeutic Applications: Development of a GMP-Grade Manufacturing Method
Source: Front Physiol. 2018 Aug 24;9:1169. doi: 10.3389/fphys.2018.01169 (PMC6117231; doi:10.3389/fphys.2018.01169)
Supplement: Supplementary file 1 [file Table_1.DOCX]

Supplementary Table 1 | Surface markers detected by the MACSPlex Exosomes Kit.

| 1 | CD3 |
| --- | --- |
| 2 | CD4 |
| 3 | CD19 |
| 4 | CD8 |
| 5 | HLA-DRDPDQ |
| 6 | CD56 |
| 7 | CD105 |
| 8 | CD2 |
| 9 | CD1c |
| 10 | CD25 |
| 11 | CD49e |
| 12 | ROR1 |
| 13 | CD209 |
| 14 | CD9 |
| 15 | SSEA-4 |
| 16 | HLA-ABC |
| 17 | CD63 |
| 18 | CD40 |
| 19 | CD62P |
| 20 | CD11c |
| 21 | CD81 |
| 22 | MCSP |
| 23 | CD146 |
| 24 | CD41b |
| 25 | CD42a |
| 26 | CD24 |
| 27 | CD86 |
| 28 | CD44 |
| 29 | CD326 |
| 30 | CD133/1 |
| 31 | CD29 |
| 32 | CD69 |
| 33 | CD142 |
| 34 | CD45 |
| 35 | CD31 |
| 36 | REA control |
| 37 | CD20 |
| 38 | CD14 |
| 39 | mIgG1 control |
