# Supplementary material for: Exosomes From Human Cardiac Progenitor Cells for Therapeutic Applications: Development of a GMP-Grade Manufacturing Method
Source: Front Physiol. 2018 Aug 24;9:1169. doi: 10.3389/fphys.2018.01169 (PMC6117231; doi:10.3389/fphys.2018.01169)
Supplement: Supplementary file 2 [file Data_Sheet_1.pdf]

ANALYTICAL METHODS

CPC count

For cell counting on frozen MCB, PPCB and EPC, vials were transferred from liquid nitrogen to a 37°C bath until thawing; 10 µL of CPC sample were immediately mixed with 10 µL of 0.4% trypan blue. 10 µL of the sample mixture were loaded on EVE™ Cell counting slide and inserted on the EVE™ Automated Cell Counter (NanoEnTek Inc., USA). This instrument provided number of total, viable and dead cells and % of viability. This procedure was repeated three times and data are shown as mean. The same counting system was used also for counting CPC during culture passages.

CPC immunophenotype analysis

The MSC Phenotyping Kit human (Miltenyi Biotec GmbH, Germany) was used. Two aliquots of CPC (from $3x10^5$ to $5x10^5$ cells) were centrifuged at 300 g for 10 min. Pellets were resuspended in 100 µl of buffer [DPBS (Gibco/Thermo Fisher Scientific, USA) with 0.5 % bovine serum albumin (BSA, Sigma-Aldrich/Merck, USA) and 2mM EDTA (Sigma-Aldrich/Merck)] and stained with 10 µl of the MSC Phenotyping Cocktail (containing antibodies specific for CPC markers CD73-APC, CD90-FITC, CD105-PE and PerCP-conjugated antibodies specific for haematopoietic markers CD14, CD20, CD34, CD45) plus 3 µl of the anti-human HLA-DR PerCP (clone AC122; Miltenyi Biotec GmbH), or 10 µl of the MSC Isotype Control Cocktail, respectively. Cells were mixed and incubated for 10 min in the dark at 4°C, washed, resuspended in 200 µl of buffer and analysed at the MACSQuant Analyzer (Miltenyi Biotec GmbH). Gates are selected to have 0.3% of positive cells in the Isotype Controls.

CPC apoptosis analysis

The BD Pharmingen™ Annexin V FITC apoptosis detection kit (Becton Dickinson, USA) was used according to manufacturer's instructions. Fluorescence signals for Annexin V (binding to the phospholipid phosphatidylserine exposed on apoptotic cell surface) and Propidium Iodide (PI, vital dye excluded by intact cell membranes) were determined by flow cytometry (CytoFLEX, Beckman Coulter, USA).

CPC RT-PCR

The following primers were used:

- GATA4 (Gene ID: 2626): GCATCAACCGGCCGCTCATCA and GGTTCTTGGGCTTCCGTTTTCT, amplicon size: 228 bp
- TBX5 (Gene ID: 6910): TACCACCACACCCATCAAC and ACACCAAGACAGGGACAGAC, amplicon size: 140 bp
- TBX18 (Gene ID: 9096): GGGTTTGGAAGCCTTGGTGG and GGCAGAATAGTCAGCAGGGG, amplicon size: 250 bp
- MESP1 (Gene ID: 55897): CTCTGTTGGAGACCTGGATG and CCTGCTTGCCTCAAAGTG, amplicon size: 278 bp
- GAPDH (Gene ID: 2597): ATGGGCAAGGTGAAGGTCGGAG and TCGCCCGACTTGATTTTGCAGG, amplicon size: 266 bp

The RNA was extracted from CPC with the GeneJet RNA Purification Kit (Thermo Fisher Scientific) and quantified by 260 nm absorbance (Infinite F-200 PRO, Tecan, Switzerland). About 500 ng of RNA per sample were reverse transcribed with the GoScript™ Reverse Transcription System kit (Promega Corporation, USA). A Reverse Transcription negative control sample was also run for each sample. Around 25 ng of cDNA-equivalent for each gene was then amplified with the GoTaq® DNA Polymerase kit (Promega Corporation) on a Thermocycler C1000 (BioRad Laboratories, USA); the

thermal profile was the following: 1x 95°C for 2 min; 32x 95°C for 0.5 min, 60°C for 0.5 min, 72°C for 0.5 min. PCR products were separated on a 2% agarose gel in Tris-acetate 1x buffer. Gel pictures were acquired by use of a Gel-Doc EZ Imaging System (BioRad Laboratories) with Image Lab™ Software, and the "Invert image display" option was applied to obtain dark bands on a light background.

Nanoparticle Tracking Analysis (NTA)

Exo-CPC samples were diluted so to obtain a final nanoparticle concentration in the range of $1x10^8$ to $2x10^9$ /ml, that is in the linear range of the instrument. Videos of this dilutions were acquired on the instrument NanoSight LM10 (Malvern Instruments, UK) with the camera gain set to 350. The videos were then analyzed by the software NTA (version 2.3, build 0034) with the minimal expected particle size, minimum track length, and blur setting, all set to automatic; the detection threshold was set to 3 to reveal all particles. The ambient temperature was recorded manually and input in the software when required. The particle concentration and the distribution graph of the particle size were determined by averaging the results from the analysis of 4 to 5 independent videos per sample; average values with the standard errors were automatically generated by the software.

Measurement of total protein content

The QuantiPro™ BCA Assay kit (Sigma-Aldrich/Merck) was used for total protein quantification; the 560 nm absorbance was determined by the Infinite F-200 PRO plate reader. The samples were undergoing 3 freeze / thaw cycles to break Exo structure immediately before running the assay. Each sample, diluted in water, was compared in duplicates against serially diluted BSA as standard. Values were extrapolated from this curve, using a linear interpolation.

Measurement of TSG101 content

TSG101 content was measured using the Human TSG101 ELISA Kit (Sandwich ELISA) - LS-F8581 from LSBio LifeSpan Biosciences Inc., USA. The samples were undergoing 3 freeze / thaw cycles to break Exo structure immediately before running the assay. All samples and standards were measured in duplicate (405 nm absorbance, determined by the Infinite F-200 PRO plate reader); the samples were appropriately diluted in the sample dilution buffer of the kit to obtain absorbance values inside the range of the standard curve (from 0.313 to 20 ng/ml). Raw absorbance data, after logarithmic transformation, were used to plot the standard curve and to determine the concentration of the TSG101 protein in the unknown samples.

Flow cytometry analysis of exosome surface markers

To characterize Exo surface markers, the MACSPlex Exosome Kit (Miltenyi Biotec GmbH) was used following manufacturer's instructions. Briefly, 6.5 ng TSG101 of Exo were stained with the capture beads (composed of 39 different bead populations differentially labelled with FITC and PE and single coated with antibodies specific for 37 different antigens or with 2 isotype control, see Supplementary Table 1) and the CD9/CD63/CD81 detection antibodies for 1 hour on an orbital shaker (450 rpm). For flow cytometry analysis, the MACSQuant Analyzer was used. Triggers for the side scatter and the forward scatter were selected to confine the measurement on the multiplex beads. Voltages for the FITC and PE channels were adapted to ensure that each of the differentially labelled bead populations were detectable. The 39 single bead populations were gated to allow the determination of the APC signal intensity on the respective bead population. Background signals were determined by analysing beads incubated only with detection antibodies and subtracted from the signals obtained for beads incubated with Exo and detection antibodies. As recommended by the MACSPlex Exosome Kit protocol, signals were normalized to the mean signal intensity obtained with the anti-CD9, CD63 and CD81 beads. A selection of markers with a higher APC fluorescence than isotype controls in at least one tested sample is shown.

Transmission electron microscopy – negative staining

Sample aliquots (7 µl) were absorbed on glow-discharged carbon-coated formvar nickel grids for 5 min. The drops were then blotted with filter paper and negatively stained with 2% uranyl acetate (5 µl) in aqueous suspension for 2 min. Excess of uranyl was removed by touching the grid to a filter paper. The grids were dried at room temperature and examined by Talos L 120C electron microscope at 120 kv.

Protein isolation and Western analyses

Total proteins were extracted by lysing Exo or cells with ice-cold RIPA buffer (SIGMA) supplemented with protease inhibitors (SIGMAFAST™ Protease Inhibitor Tablets; Sigma) for 30 min at 4°C under agitation. Protein concentrations were determined using the BCA kit (Thermo Fisher). Proteins were boiled with Laemmli SDS sample buffer 6X (VWR International LCC), separated on 4–20% Mini-PROTEAN® TGX™ Precast Gel (Bio-Rad), and transferred onto a PVDF membrane with a semi-dry transfer system (BioRad). The membranes were blocked for 1 hr in Odyssey Blocking Buffer (LI-COR Biosciences, USA), then incubated with the appropriate primary Ab (anti-Periostin mouse monoclonal Ab, Santa Cruz, UK; anti-TSG101 rabbit monoclonal Ab, anti-GRP94 rabbit polyclonal Ab, anti-GAPDH rabbit monoclonal Ab, Abcam, UK) diluted 1:1000in OBB-T at 4°C overnight under gentle agitation. Membranes were rinsed and then incubated with a IRDye® 680RD or 800CW goat anti-mouse or goat anti-rabbit secondary Ab (LI-COR Biosciences; 1:12500 dilution in OBB-T) at RT for 2 hrs. The infrared signal was detected using the Odyssey CLx Detection System (LI-COR Biosciences).

Sterility Test

The sterility test was carried out on MCB, PPCB, EPC and GMP-Exo-CPC by inoculating a representative sample volume (500 µL) in Bact/Alert FA PLUS (aerobic microorganism growth) and BACT/Alert FN PLUS (anaerobic microorganism growth) bottles (bioMérieux, France). The two bottles were incubated into the BACT/ALERT 3D (bioMérieux) for 14 days.

Bacterial endotoxins Test

A colorimetric Limulus Amebocyte Lysate test was used to quantify endotoxins from gram-negative bacteria, according to endosafe®-PTS™ system (Charles River, USA). To this purpose, Endosafe-Licensed PTS Endotoxin Cartridges (Charles River) with 0.05 EU/ml sensitivity were loaded with 25ul /well of sample diluted 1:50 in sterile water. Cartridges were analyzed by the Endosafe-PTS (Charles River), a spectrophotometric reader with an incubation chamber (37°C) that allows the detection of the signal generated by the chromogenic substrate present in the cartridges.

The test was anticipated by the Inhibition/Enhancement test to define the appropriate sample dilution to avoid interference phenomena.

Exosome functional assays

The anti-apoptotic activity of Exo was tested by an *in vitro* apoptosis/viability assay developed in house. Human CPC or Mouse HL-1 cardiomyocytic cells were plated (10^4 cells/well) in black 96 well plate with transparent flat bottom (Corning) in complete medium, upon coating with 0.2% gelatin (Sigma-Aldrich/Merck) for 30 min at 37°C. Complete medium for CPC is StemMACS™ MSC expansion Media kit XF (Miltenyi Biotec GmbH) while Complete medium for HL-1 is Claycomb Medium (Sigma-Aldrich, St. Louis, MO, USA) supplemented with 100 µM norepinephrin, 10% FBS, 1% v/v penicillin streptomycin and 4 mM L-glutamine (all from Gibco, Life Technologies, Carlsbad, CA, USA). The day after, cells were treated with 1µM staurosporine (Sigma-Aldrich/Merck) in basal medium

(DMEM without glucose and L-glutamine, GE Healthcare Life Sciences, UK, for CPC and Claycomb Medium with no supplements for HL-1) with or without Exo. Some wells were maintained in complete medium as a control of cell growth; 10% FBS was added to basal medium as positive control for protection and wells without cells were prepared as blanks. Exo were added at different concentrations, relying on the particles number or TSG101 quantification, and equal volumes of vehicle (Plasma-Lyte A® solution, Baxter Healthcare, USA) and/or Exo from normal human dermal fibroblasts (Exo-F) were used as controls. After overnight incubation at 37°C, 5% CO_2, cell death was quantified by staining with 2.5 μM Propidium Iodide (PI, Thermo Fisher Scientific) for 30 min at 37°C, followed by fluorescence detection with the Infinite F-200 PRO plate reader. After a washing with DPBS, images of cells were taken with a 4x and a 10x magnification. Cells were then stained with 3 μM calcein AM (Thermo Fisher Scientific) for 30 min at 37°C and viability was quantified by fluorescence detection with the Infinite F-200 PRO plate reader. Fluorescence of blank wells was subtracted from the fluorescence obtained from respective sample wells. Apoptosis data were normalized on 1μM staurosporine basal medium condition, while viability data were normalized on 1μM staurosporine complete medium condition.

The pro-angiogenic activity of Exo was tested by the V2a Kit (Cellworks, UK), an *in vitro* test designed to determine angiogenic activity of compounds or conditioned media. In the patented Cellworks V2a Kit, human umbilical vein endothelial cells are co-cultured with human dermal fibroblasts in a specially designed medium. The endothelial cells initially form small islands within the culture matrix. They subsequently begin to proliferate and then enter a migratory phase during which they move through the matrix to form threadlike tubule structures. These gradually join up (by 1 - 2 weeks) to form a network of anastomosing tubules which closely resembles the capillary bed found in the chicken embryo chorioallantoic membrane assay. The tubules stain positive for von Willebrand's Factor, PECAM 1 (CD31) and ICAM-2. We tested Exo at different concentrations (3 and 15 ng of TSG101/ml) and used same volumes of vehicle as control. The angiogenic stimulator VEGF and the inhibitor Suramin were included as positive and negative controls, respectively. Complete medium was also used as control of the basal proliferation effect. Media were changed every 48-72 hours and after 12-14 days cells were fixed in cold ethanol 70% (Sigma-Aldrich/Merck) and subsequently stained by a tubule staining kit containing anti-CD31 primary antibody and a secondary antibody conjugated with alkaline phosphatase (AP). The level of angiogenesis was detected by two means:

1) CD31 ELISA Soluble Substrate: SIGMAFAST™ p-Nitrophenyl phosphate Tablets (Sigma-Aldrich/Merck) were dissolved in water according to manufacturer's instructions and added to stained cells. p-Nitrophenyl phosphate (p-NPP) is hydrolyzed by AP in ρ-Nitrophenol, a chromogenic product that absorbs maximally at 405 nm. After 20 min at 37°C, two 100 μl aliquots were taken from each well and plated in a 96 well plate containing 25 μl of 3M NaOH (Sigma-Aldrich/Merck). Blank samples were also prepared by adding p-NPP directly to NaOH. Absorbance at 405 nm of sample wells was measured against the blank wells by the Infinite F-200 PRO plate reader. This read out not included in the V2a Kit was implemented to have a more quantitative and standardized measure of angiogenetic activity of Exo.

2) CD31 Tubule Staining Insoluble Substrate: cells were washed three times with 0.5 ml of deionized water and stained with BCIP/NBT according to manufacturer's instructions (V2a Kit, Cellworks). Tubule formation was visualized by 4x magnification optical microscope and analyzed by the AngioSys 2.0 Image Analysis Software (Cellworks), that provides a semi-automated analysis of angiogenesis by measuring the number of tubules, the number of junctions, the total tubule length, and the mean tubule length for each image. This software has been specifically developed to quantitate images of angiogenesis tubules obtained from the V2a Kit. For each treatment condition, mean of total tube length from 3 wells was plotted.

SUPPLEMENTARY FIGURE LEGENDS

Supplementary Figure 1. Western Blot analysis of Exo. TSG-101: specific Exo marker; Periostin: protein enriched in CPC-derived EV; GRP94: cell marker, used as indicator of cell contaminants from cell culture; GAPDH: reference marker.

Supplementary Figure 2. Flow cytometric characterization of Exo-CPC-I, Exo-CPC-II and Exo-CPC-R. Exo-CPC were analysed with the MACSPlex Exosome Kit. Data are represented as APC mean fluorescence intensity normalized to the mean signal intensity obtained with the anti-CD9, anti-CD63 and anti-CD81 beads

Supplementary Figure 3. *In vitro* anti-apoptotic activity of Exo on CPC and HL-1 cells. Apoptotic agent: 1μM staurosporine. CRL + = 10% FBS; Exo-CPC-I dose: 0.25 ng of TSG101/ml; Vehicle = Plasma-Lyte A®. Vehicle was added at the same volume as Exo-CPC-I. (**A**) Results from calcein staining on HL-1 cells. (**B**) Results from calcein staining on CPC cells. The treatment with complete medium + staurosporine is the 100% survival reference. Bars: mean + SD (n = 5). *** = p<0.001 One way ANOVA with Student's t-test with Bonferroni correction.

Supplementary Figure 4. Characterization of cells during starvation for Exo production. (**A**) Representative images of confluent CPC-I before starvation (PRE) and at different times during starvation (POST 1 week and POST 2 weeks). (**B**) FACS analysis. Expression of surface markers on CPC-I at different times during starvation. (**C**) Apoptosis analysis. Annexin V/PI staining on cells at day 12 during starvation. Viable cells = Annexin V negative/PI negative; early apoptotic cells = Annexin V positive/PI negative; late apoptotic/necrotic = Annexin V positive/PI positive plus Annexin V negative/PI positive.

Supplementary Figure 5. Flow cytometric characterization of GMP-Exo-CPC. Exo-CPC were analysed with the MACSPlex Exosome Kit. Data are represented as APC mean fluorescence intensity.
