# Supplementary figures and images for: Exosomes From Human Cardiac Progenitor Cells for Therapeutic Applications: Development of a GMP-Grade Manufacturing Method
Source: Front Physiol. 2018 Aug 24;9:1169. doi: 10.3389/fphys.2018.01169 (PMC6117231; doi:10.3389/fphys.2018.01169)

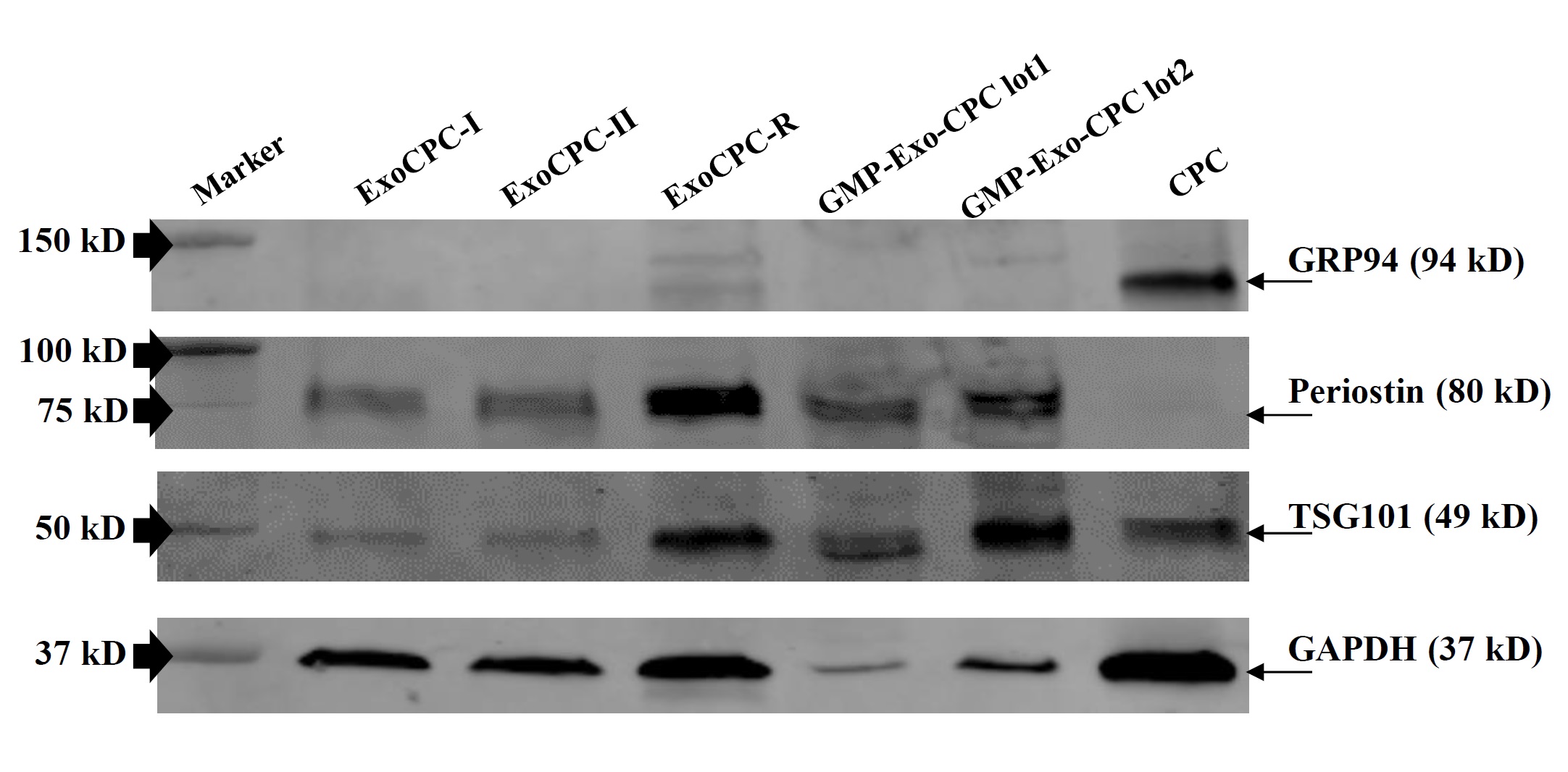

Supplement: Supplementary file 3 [file Image_1.JPEG]

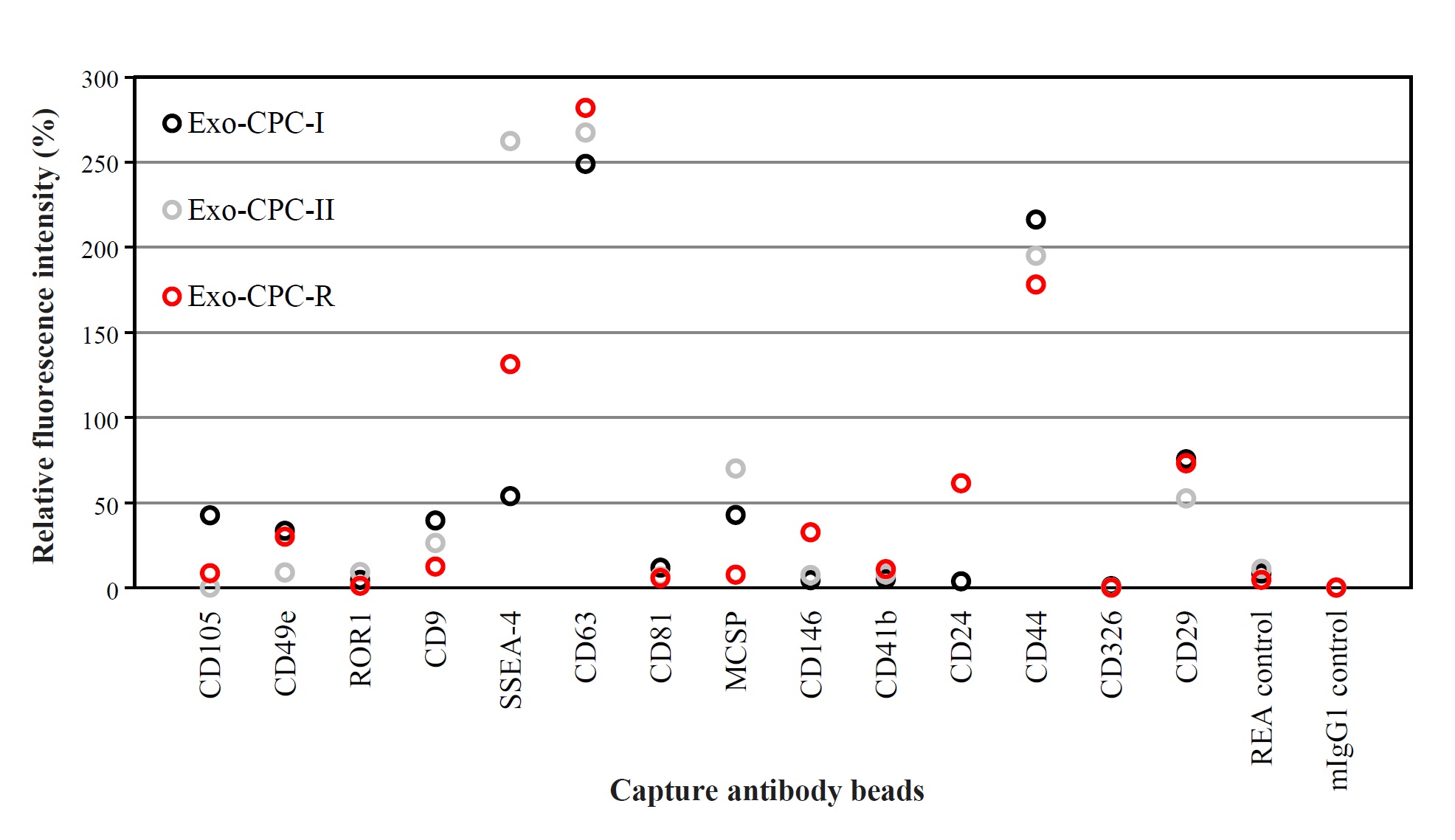

Supplement: Supplementary file 4 [file Image_2.JPEG]

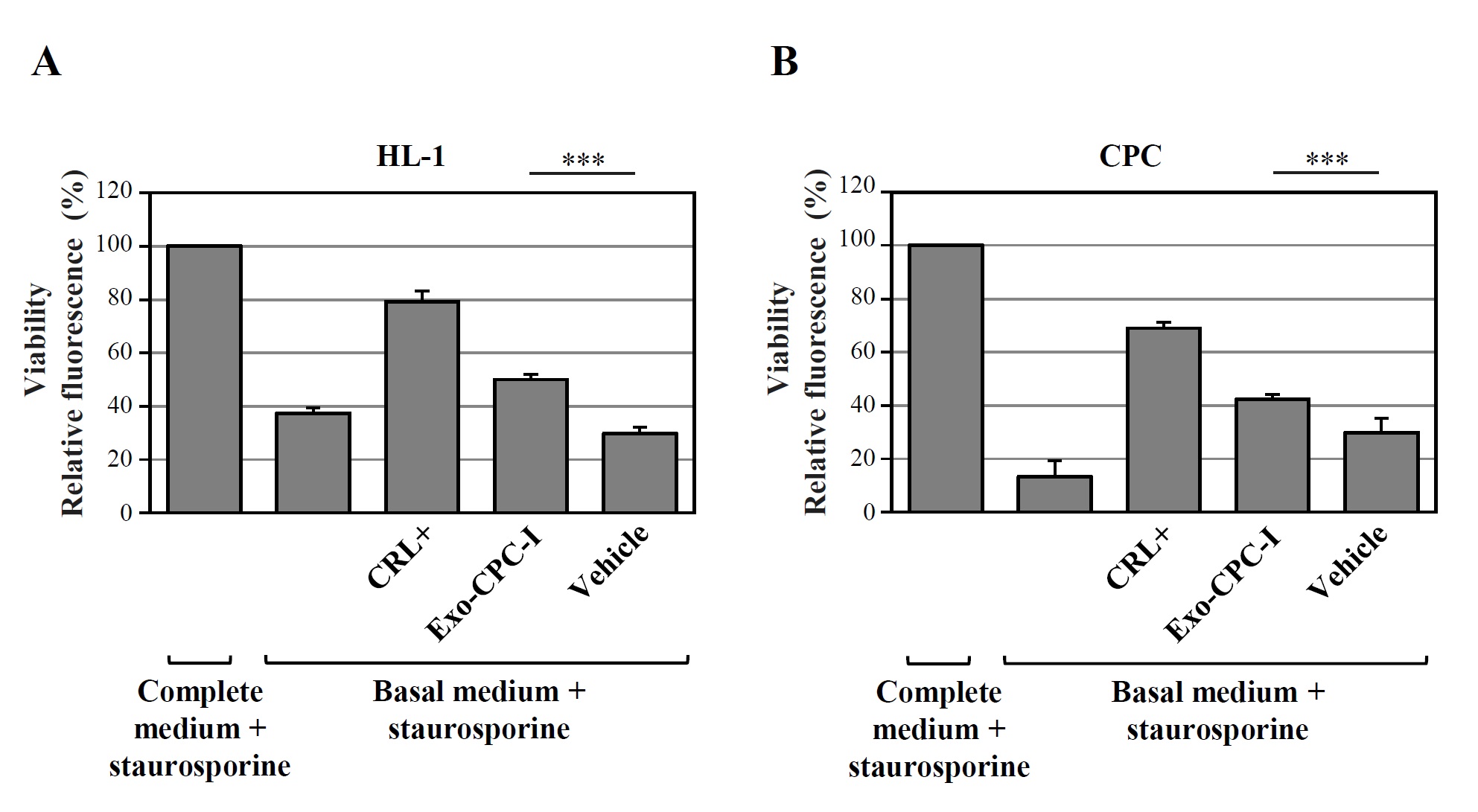

Supplement: Supplementary file 5 [file Image_3.JPEG]

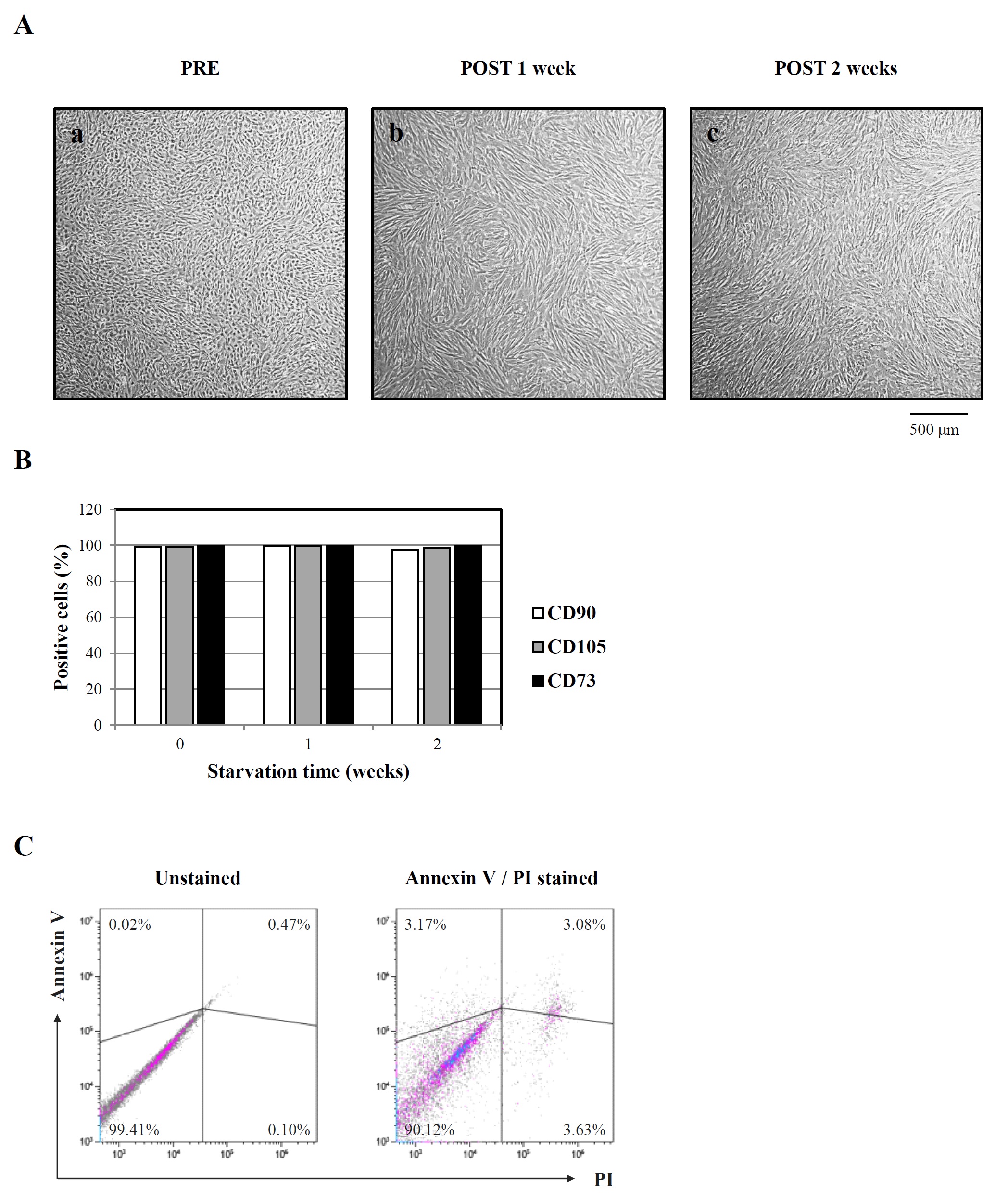

Supplement: Supplementary file 6 [file Image_4.JPEG]

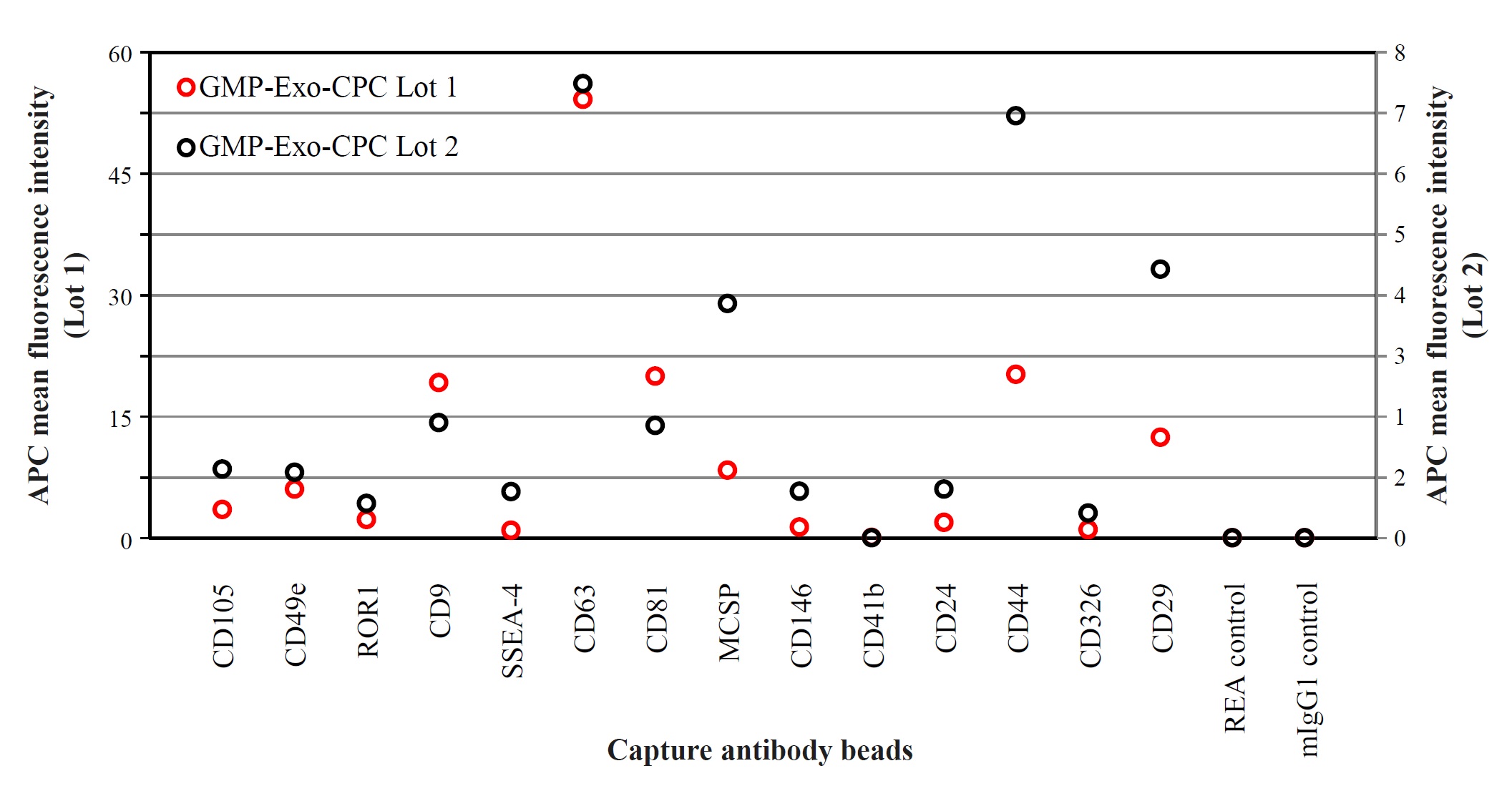

Supplement: Supplementary file 7 [file Image_5.JPEG]
